# Instanton theory for Fermi’s golden rule and beyond

**DOI:** 10.1098/rsta.2020.0378

**Published:** 2022-05-16

**Authors:** Imaad M. Ansari, Eric R. Heller, George Trenins, Jeremy O. Richardson

**Affiliations:** Laboratory of Physical Chemistry, ETH, Zürich, Switzerland

**Keywords:** fourth-order rate, non-adiabatic, tunnelling, bridge-mediated electron transfer, superexchange

## Abstract

Instanton theory provides a semiclassical approximation for computing quantum tunnelling effects in complex molecular systems. It is typically applied to proton-transfer reactions for which the Born–Oppenheimer approximation is valid. However, many processes in physics, chemistry and biology, such as electron transfers, are non-adiabatic and are correctly described instead using Fermi’s golden rule. In this work, we discuss how instanton theory can be generalized to treat these reactions in the golden-rule limit. We then extend the theory to treat fourth-order processes such as bridge-mediated electron transfer and apply the method to simulate an electron moving through a model system of three coupled quantum dots. By comparison with benchmark quantum calculations, we demonstrate that the instanton results are much more reliable than alternative approximations based on superexchange-mediated effective coupling or a classical sequential mechanism.

This article is part of the theme issue ‘Chemistry without the Born–Oppenheimer approximation’.

## 1. Introduction

The Born–Oppenheimer approximation is widely used to simplify the Schrödinger equation for molecular systems by expressing the equations of motion in terms of a single adiabatic potential-energy surface (PES). However, there are many molecular processes for which this adiabatic approximation is not valid, typically due to two PESs becoming close in energy. In these situations, alternative approaches are necessary.

One way of going beyond the Born–Oppenheimer approximation is to represent the total Hamiltonian in the basis of diabatic electronic states [1]. If the coupling between the diabatic states is small, perturbation theory can be used to obtain solutions correct to a given order. Typically, the leading-order approximation is second order in the coupling, and the resulting expression is commonly called Fermi’s Golden Rule (FGR) [2]. Within this theory, the rate for a transition from an initial state i to a final state f is [3–5]

1.1 $$k_{if}^{\left(2\right)}=\frac{2\pi }{\hbar }|\Delta_{if}{|}^{2}\delta (E_{f}-E_{i}),$$

which is proportional to the square of the coupling between the states, $\Delta_{if}$, and by virtue of the delta function, enforces energy conservation. This expression can alternatively be written in terms of quantum-mechanical propagators or Green’s function operators [6,7] and is also known as ‘the first Born approximation’ in scattering theory [8].

FGR is very general and can in principle be applied to study any physical system that evolves between a pair of weakly coupled initial and final states. It has found applications in various fields, including spectroscopy [9,10], scattering processes such as X-ray diffraction [6,8], scanning tunnelling microscopy [11], and the kinetics of nuclear [2] and electron-transfer [12,13] reactions.

Marcus theory can be recovered from equation (1.1) by taking into account the initial thermal distribution of particles and replacing the quantum states with a classical phase-space average under the assumption of parabolic free-energy surfaces [14]. An important consequence of this theory was the prediction and later experimental verification of the inverted regime [15]. However, these classical theories neglect quantum tunnelling, and many quantum-mechanical methods developed to extend Marcus theory rely on the assumption of harmonic PESs, for which the analytic solutions to the Schrödinger equation are known [12]. These approaches thus constitute uncontrolled approximations which may not always reflect the reality of complex anharmonic molecular systems. Instead, we require a formalism which provides a rigorous asymptotic approximation to the FGR rate without the necessity of first solving the Schrödinger equation, even for the reference Hamiltonians.

Semiclassical instanton (SCI) theory [16,17] provides a framework for describing multidimensional tunnelling effects in complex molecular systems. It is based on the path-integral formulation of quantum mechanics [6] but avoids summing over all possible paths by identifying the optimal tunnelling pathway, which is called the ‘instanton’. The instanton pathway is defined as the path which makes the action stationary. It is represented by a periodic classical orbit in imaginary time, or equivalently one which obeys Newton’s equations of motion on an upside-down PES [16]. The use of imaginary time to simulate tunnelling processes can be justified by considering a particle crossing a potential-energy barrier, V(x), with energy lower than the barrier top. In order to pass through the region where E<V(x), the kinetic energy must take negative values, which implies that the particle will have imaginary momentum and can therefore be said to travel in imaginary time.

The ring-polymer formulation of instanton theory provides computationally efficient algorithms for locating the optimal tunnelling pathways on a single Born–Oppenheimer PES and evaluating the rate [17–22]. Because the method is based on a unique optimal pathway and does not rely on sampling a statistically large number of path configurations, it can be applied to complex molecular systems which require expensive electronic-structure calculations.^1^ In addition to calculating rates accurately, the instanton pathway also offers clear mechanistic insight into the tunnelling process. For instance, quantitative information on the contribution from each atom can be extracted such that kinetic isotope effects can be easily predicted and understood [26–28]. It has, for instance, been applied to gas-phase reactions [29–31], tunnelling in molecules and clusters [28,32–35], enzymatic reactions [36], surface diffusion processes [37,38] and water formation reactions [39].

An extension of the original instanton theory has been derived which provides a semiclassical approximation to FGR rates of non-adiabatic reactions and predicts very good results for benchmark model systems [40–42]. Unlike the Zhu–Nakamura formulae [43] (which extend Landau–Zener theory [44,45] to include tunnelling along a one-dimensional reaction coordinate), instantons constitute a multidimensional tunnelling theory. They are thus expected to provide more accurate results than the one-dimensional approaches (assuming the reaction takes place in the golden-rule limit), although at an enhanced computational cost as information is required along the instanton path and not just at a single point. The ring-polymer discretization of the instanton trajectory again provides a computationally efficient algorithm in both the normal [46,47] and inverted regimes [42], and we present the working equations in §3.

For many chemical processes, however, it is necessary to go beyond the golden-rule approximation. This is particularly clear for systems where the initial and final states are both coupled to a third ‘bridge’ state but not to each other. This process is sometimes referred to as the superexchange mechanism [48–50] or the through-bond coupling mechanism [51]. Examples include bridge-mediated electron transfer in bacterial photosynthetic reaction centres [52], hole migration in DNA [50], CO-haemoglobin recombination [12] and two-photon processes such as Raman spectroscopy [10]. Such processes are described by higher-order perturbation-theory, leading to the fourth-order rate constant [5]

1.2 $$k_{if}^{\left(4\right)}=\frac{2\pi }{\hbar }{\left|\sum_{b}\frac{\Delta_{ib}\Delta_{bf}}{E_{b}-E_{i}}\right|}^{2}\delta (E_{f}-E_{i}).$$

This describes a concerted mechanism from i to f via intermediate bridge states b [49].

Just as for the second-order FGR case, equation (1.2) can only be evaluated if one first solves the time-independent Schrödinger equation for the uncoupled problem in order to obtain the energy levels i, f and b. For complex molecular systems, this is practically impossible without making further approximations. Even if reasonable approximations are obtained for the energy levels, the calculation of the sum over the intermediate b states can be challenging. This is because the individual terms in the sum, $\Delta_{ib}\Delta_{bf}$, may be either positive or negative, and a large number of the terms are similar in magnitude but differ in the sign. This makes it difficult both to evaluate the sum numerically and to extract a simple mechanistic interpretation from the expression.

In this paper, we derive a new fourth-order instanton approximation as an extension of the derivation in the golden-rule limit. This provides reasonably accurate numerical results as well as a simple mechanistic interpretation for the reaction. We start by reviewing the existing golden-rule framework before tackling the fourth-order case in §4.

## 2. Golden-rule instanton theory

In this section, we outline the derivation of golden-rule instanton theory starting from the standard expression for Fermi’s golden rule.

### (a) Fermi’s golden rule

We consider the total Hamiltonian

2.1 $$\hat{H}={\hat{H}}_{R}|R\rangle \langle R|+{\hat{H}}_{P}|P\rangle \langle P|+{\hat{\Delta }}_{\mathrm{RP}}(|R\rangle \langle P|+|P\rangle \langle R|),$$

where |R⟩ and |P⟩ are the diabatic electronic states of the reactant and product.

The uncoupled Hamiltonians are typically given in terms of the PESs, $V_{n}(x)$, by

2.2 $${\hat{H}}_{n}=\frac{{\hat{p}}^{2}}{2m}+V_{n}(\hat{x}),$$

where n∈{R,P}. This describes a system with f nuclear degrees of freedom with coordinates x and conjugate momenta p which have been mass-weighted such that the associated mass of each degree of freedom is m. The electronic states are coupled by ${\hat{\Delta }}_{\mathrm{RP}}=\Delta_{\mathrm{RP}}(\hat{x})$, which is assumed to be small and slowly varying.

In the standard perturbation theory approach, we assume that we can solve the time-independent Schrödinger equation for the unperturbed Hamiltonians

2.3 $${\hat{H}}_{R}|\chi_{R}^{\mu }\rangle =E_{R}^{\mu }|\chi_{R}^{\mu }\rangle \;\mathrm{and}\;{\hat{H}}_{P}|\chi_{P}^{\nu }\rangle =E_{P}^{\nu }|\chi_{P}^{\nu }\rangle .$$

Each set of vibrational states (with quantum numbers μ or ν) forms a complete basis of the nuclear Hilbert space such that $1=\sum_{\mu }|\chi_{R}^{\mu }\rangle \langle \chi_{R}^{\mu }|=\sum_{\nu }|\chi_{P}^{\nu }\rangle \langle \chi_{P}^{\nu }|$. The vibronic states $|\chi_{R}^{\mu }\rangle ⊗|R\rangle$ and $|\chi_{P}^{\nu }\rangle ⊗|P\rangle$ form a basis for the total Hamiltonian and are coupled by $\Delta_{\mathrm{RP}}^{\mu \nu }=\langle \chi_{R}^{\mu }|{\hat{\Delta }}_{\mathrm{RP}}|\chi_{P}^{\nu }\rangle$.

From equation (1.1), taking a Boltzmann average over the initial vibronic states $|\chi_{R}^{\mu }\rangle ⊗|R\rangle$, for which the reactant partition function is $Z_{R}=\mathrm{Tr}[e^{-\beta {\hat{H}}_{R}}]$, and summing over all final states $|\chi_{P}^{\nu }\rangle ⊗|P\rangle$, the second-order thermal rate at inverse temperature $\beta ={\left(k_{B}T\right)}^{-1}$ is given by $k^{\left(2\right)}$, where

2.4*a* $$\begin{array}{rl} k^{\left(2\right)}Z_{R} & \;=\frac{2\pi }{\hbar }\sum_{\mu }e^{-\beta E_{R}^{\mu }}\sum_{\nu }|\Delta_{\mathrm{RP}}^{\mu \nu }{|}^{2}\delta (E_{R}^{\mu }-E_{P}^{\nu }) \end{array}$$

2.4*b* $$\begin{array}{rl} & \;=\frac{1}{\hbar^{2}}\int_{-\infty }^{\infty }\sum_{\mu \nu }e^{-[(\beta \hbar -\tau )E_{R}^{\mu }+\tau E_{P}^{\nu }]/\hbar }|\Delta_{\mathrm{RP}}^{\mu \nu }{|}^{2}\;e^{i(E_{R}^{\mu }-E_{P}^{\nu })t/\hbar }\;dt. \end{array}$$

To derive equation (2.4b), we first replaced $\beta \hbar E_{R}^{\mu }$ by $(\beta \hbar -\tau )E_{R}^{\mu }+\tau E_{P}^{\nu }$. This is valid for any value of τ due to the presence of the delta function, which forces the two energies to be equal. The reason for this manipulation will become apparent later. Next, we replaced the delta function by its Fourier integral representation. This allows us to rewrite the rate expression in a basis-independent form without reference to the eigenstates of the uncoupled systems and paves the way for a path-integral formulation. The quantum-mechanical rate can thus be defined by

2.5 $$k^{\left(2\right)}Z_{R}=\frac{1}{\hbar^{2}}\int_{-\infty }^{\infty }c^{\left(2\right)}(\tau +it)\;dt,$$

with the correlation function

2.6 $$c^{\left(2\right)}(\tau +it)=\mathrm{Tr}[{\hat{\Delta }}_{\mathrm{RP}}^{†}\;e^{-(\beta \hbar -\tau -it){\hat{H}}_{R}/\hbar }\;{\hat{\Delta }}_{\mathrm{RP}}\;e^{-(\tau +it){\hat{H}}_{P}/\hbar }].$$

This is related to the general flux correlation function formulation of rate theory [53–55] (although subtly different due to the asymmetric shift introduced by τ [18,24]) and is the starting point for a number of path-integral simulations of the golden-rule rate [25,41,46,56–60]. Note that $c^{\left(2\right)}(z)$ can be thought of as an analytic function of z=τ+it and that modifying τ is thus equivalent to deforming the integration contour in the complex plane. Thus, although $c^{\left(2\right)}(\tau +it)$ explicitly depends on τ, the Cauchy integral theorem [61] ensures that $k^{\left(2\right)}$ is independent of τ, which will allow us in the following sections to pick the most convenient value.

### (b) Semiclassical instanton theory

We have shown how the FGR rate can be expressed in terms of the second-order correlation function, $c^{\left(2\right)}(\tau +it)$. In this subsection, we derive an SCI approximation to the correlation function and hence to the FGR rate itself.

First, we write the correlation function in the basis of position states

2.7 $$c^{\left(2\right)}(\tau +it)=\iint \Delta_{\mathrm{RP}}^{*}(x^{\prime })K_{R}(x^{\prime },x^{\prime \prime },\beta \hbar -\tau -it)\Delta_{\mathrm{RP}}(x^{\prime \prime })K_{P}(x^{\prime \prime },x^{\prime },\tau +it)\;dx^{\prime }\;dx^{\prime \prime },$$

where the quantum-mechanical propagators, $K_{n}(x^{\prime \prime },x^{\prime },z)=\langle x^{\prime \prime }|e^{-z{\hat{H}}_{n}/\hbar }|x^{\prime }\rangle$, give the probability amplitude for a particle starting at $x^{\prime }$ to travel to $x^{\prime \prime }$ in complex time z (the real part of z corresponds to imaginary time and the imaginary part to real time). We will see later that the time integral over the correlation function is dominated by its value at t=0, and thus the propagators can be approximated using the imaginary-time generalization [62] of the van-Vleck semiclassical propagator [63]

2.8 $$K_{n}(x^{\prime \prime },x^{\prime },\tau_{n})∼\sqrt{\frac{C_{n}}{{\left(2\pi \hbar \right)}^{f}}}\;e^{-S_{n}(x^{\prime \prime },x^{\prime },\tau_{n})/\hbar }.$$

Here, the action is defined as

2.9 $$S_{n}(x^{\prime \prime },x^{\prime },\tau_{n})=\int_{0}^{\tau_{n}}[\frac{1}{2}m||\dot{x}(u)|{|}^{2}+V_{n}(x(u))]\;du,$$

for the imaginary-time classical trajectory x(u) which solves $m\ddot{x}=\nabla V(x)$, i.e. Newton’s equation of motion on the upside-down PES [62], with the boundary conditions $x(0)=x^{\prime }$ and $x(\tau_{n})=x^{\prime \prime }$. The prefactor is given by the determinant of the f×f second-derivative matrix of the action:

2.10 $$C_{n}=\left|-\frac{\partial^{2}S_{n}}{\partial x^{\prime }\partial x^{\prime \prime }}\right|.$$

The remaining integrals in equations (2.5) and (2.7) are evaluated using the steepest-descent approximation, which, for the general case given below, is derived by expanding the exponent to second order around its stationary point and the prefactor to lowest order,

2.11*a* $$\begin{array}{rl} \int A(z)\;e^{-S(z)/\hbar }\;dz & \;∼\int A(\tilde{z})\;e^{-\frac{1}{\hbar }S(\tilde{z})-\frac{1}{2\hbar }(z-\tilde{z})\cdot \Phi (\tilde{z})\cdot (z-\tilde{z})}\;dz \end{array}$$

2.11*b* $$\begin{array}{rl} & \;=\frac{{\left(2\pi \hbar \right)}^{d/2}}{\sqrt{\det\Phi (\tilde{z})}}A(\tilde{z})\;e^{-S(\tilde{z})/\hbar }, \end{array}$$

where z is a vector of dimension d, A(z) and S(z) are arbitrary analytic functions, $\Phi (z)=\frac{\partial^{2}S}{\partial z\partial z}$, $\tilde{z}$ is the solution of $\frac{\partial S}{\partial z}=0$ and we have assumed that $\Phi (\tilde{z})$ is positive definite and that $A(\tilde{z})\neq 0$. For our purposes, the integration range is typically over the whole real axis, but the expression is valid as long as $\tilde{z}$ is inside the range. In the following, we shall omit the tilde; whenever z appears outside an integral, we imply that it is evaluated at the stationary point $\tilde{z}$. It can be proved that the steepest-descent approximation is an asymptotic expansion of the integral in ℏ and is thus exact in the limit ℏ→0 [64]. The van-Vleck propagator itself can be derived as a steepest-descent approximation to the exact path-integral propagator [63,65].

We then arrive at the SCI approximation by replacing the propagators with their semiclassical limits (equation (2.8)) and performing the integrals over space and time simultaneously with the method of steepest descent (equation (2.11)). Typically, a stationary point can be found with real-valued $x^{\prime }$, $x^{\prime \prime }$ and τ, which corresponds to t=0. The final expression for the rate is thus

2.12 $$k_{\text{SCI}}^{\left(2\right)}Z_{R}=\sqrt{2\pi \hbar }\;\frac{|\Delta_{\mathrm{RP}}{|}^{2}}{\hbar^{2}}\sqrt{\frac{C_{R}C_{P}}{-\Sigma^{\left(2\right)}}}\;e^{-S^{\left(2\right)}/\hbar },$$

where $S^{\left(2\right)}\equiv S^{\left(2\right)}(x^{\prime },x^{\prime \prime },\tau )=S_{R}(x^{\prime },x^{\prime \prime },\beta \hbar -\tau )+S_{P}(x^{\prime \prime },x^{\prime },\tau )$ and all expressions (including $\Delta_{\mathrm{RP}}$) are evaluated with $x^{\prime }$, $x^{\prime \prime }$ and τ at the values that make the total action stationary. The prefactor includes the determinant

2.13 $$\Sigma^{\left(2\right)}=\left|\begin{array}{ccc} \frac{\partial^{2}S^{\left(2\right)}}{\partial x^{\prime }\partial x^{\prime }} & \frac{\partial^{2}S^{\left(2\right)}}{\partial x^{\prime }\partial x^{\prime \prime }} & \frac{\partial^{2}S^{\left(2\right)}}{\partial x^{\prime }\partial \tau }\\ \frac{\partial^{2}S^{\left(2\right)}}{\partial x^{\prime \prime }\partial x^{\prime }} & \frac{\partial^{2}S^{\left(2\right)}}{\partial x^{\prime \prime }\partial x^{\prime \prime }} & \frac{\partial^{2}S^{\left(2\right)}}{\partial x^{\prime \prime }\partial \tau }\\ \frac{\partial^{2}S^{\left(2\right)}}{\partial \tau \partial x^{\prime }} & \frac{\partial^{2}S^{\left(2\right)}}{\partial \tau \partial x^{\prime \prime }} & \frac{\partial^{2}S^{\left(2\right)}}{\partial \tau^{2}} \end{array}\right|,$$

and the negative sign appears as a consequence of the Cauchy–Riemann equations, $i\frac{\partial }{\partial \tau }=\frac{\partial }{\partial t}$. As we show in appendix A, an equivalent derivation also holds in the inverted regime. For consistency, we choose to evaluate the reactant partition function, $Z_{R}$, using an equivalent semiclassical limit, which typically corresponds to the standard quantum-mechanical harmonic approximation [18].

The stationary-point conditions, $\partial S^{\left(2\right)}/\partial x^{\prime }=\partial S^{\left(2\right)}/\partial x^{\prime \prime }=0$ and $\partial S^{\left(2\right)}/\partial \tau =0$, give important information about the instanton. In particular, because $\partial S^{\left(2\right)}/\partial x^{\prime }=\partial S_{R}/\partial x^{\prime }+\partial S_{P}/\partial x^{\prime }\equiv p_{R}^{\prime }-p_{P}^{\prime }$ (and equivalently with double primes), momentum is conserved at the hopping points. Also, if we identify $\tau_{R}\equiv \beta \hbar -\tau$ and $\tau_{P}\equiv \tau$, we find that because $\partial S^{\left(2\right)}/\partial \tau =-\partial S_{R}/\partial \tau_{R}+\partial S_{P}/\partial \tau_{P}\equiv -E_{R}+E_{P}$, energy must be conserved. The hopping points must therefore be located on the crossing seam, where $V_{R}(x)=V_{P}(x)$, but not necessarily at the minimum-energy crossing point (MECP).

In previous work, we have applied the instanton approach to various model systems including asymmetric system-bath models of electron transfer [47] and photodissociation in anharmonic potentials [42]. The results compared favourably with quantum-mechanical benchmarks and were seen to be much more accurate than alternative methods such as classical Marcus theory and the cumulant expansion [66].

Typically, for molecular systems in the gas phase and even many systems in the condensed phase, we have to take into account that in addition to the vibrational degrees of freedom the molecule also possesses $f_{0}$ external degrees of freedom comprising translations and/or rotations. The presence of these external degrees of freedom causes some of the eigenvalues of $\Sigma^{\left(2\right)}$ to be zero, preventing a straightforward application of the steepest descent formula. We can, however, project them out and account for these degrees of freedom by the standard expressions for the partition functions for global translation and rotation.

In order to account for the external degrees of freedom we first consider the free-particle action given by $S_{n}^{\text{free}}(X^{\prime \prime },X^{\prime },\tau_{n})=m||X^{\prime \prime }-X^{\prime }|{|}^{2}/2\tau_{n}$. Because $\partial^{2}S_{n}^{\text{free}}/\partial X^{\prime }\partial X^{\prime \prime }=-m/\tau_{n}$, it can readily be seen that translations and rotations manifest themselves in $-\partial^{2}S_{n}/\partial x^{\prime }\partial x^{\prime \prime }$ in the form of $f_{0}$ eigenvalues equal to $m/\tau_{n}$. A similar argument shows that $\Sigma^{\left(2\right)}$ has $f_{0}$ zero eigenvalues and $f_{0}$ eigenvalues equal to $2m(1/\tau_{R}+1/\tau_{P})$ resulting from the external degrees of freedom. The contributions of the translations and rotations can thus be separated by removing these eigenvalues in the calculation of the determinants $C_{R},C_{P}$ and $\Sigma^{\left(2\right)}$. Finally, under the assumption that rotations and translations do not couple to the vibrations, one multiplies the rate equation (2.12) by the translational and rotational partition function corresponding to the MECP or averaged along the instanton [18].

### (c) Application to the separable case

If we apply the instanton formulation of the previous subsection to a separable Hamiltonian, we obtain a closed-form formula for the golden-rule rate. In [41], a fully classical rate formula was presented based on the knowledge of the minimum-energy crossing point. We go a step further and employ a separable model which includes tunnelling along an arbitrary one-dimensional reaction coordinate and quantized vibrations in the perpendicular modes in an analogous way to Eyring’s transition-state theory (TST) [67]. The formula we obtain is similar but not identical to those commonly used in the literature [7,12,68–70]. The key approximation here neglects frictional effects (i.e. coupling between the reaction coordinate and the other modes) which would otherwise cause the instanton path to curve and would require a full-dimensional numerical solution as described in §3.

The most important configuration for reactions in the golden-rule limit is the minimum-energy crossing point, $x^{\text{‡}}$, defined by the minimum of $V_{R}(x)$ subject to the constraint $V_{R}(x)=V_{P}(x)$, which generalizes the transition-state concept to non-adiabatic rate theory. At this point, the potential is $V_{R}(x^{\text{‡}})=V_{P}(x^{\text{‡}})=V^{\text{‡}}$ and the two gradients, $g_{R}=\nabla V_{R}(x^{\text{‡}})$ and $g_{P}=\nabla V_{P}(x^{\text{‡}})$, are either parallel or antiparallel, corresponding to the inverted or normal regime. The Hessians are $H_{n}=\nabla \nabla V_{n}(x^{\text{‡}})$; note that in general it will not be possible to find a normal-mode transform which simultaneously diagonalizes $H_{R}$ and $H_{P}$.

The potential-energy surface is assumed to be separable around the MECP and we use a coordinate system consisting of a reaction coordinate, q, which points in the direction of the MECP gradient, $g^{\text{‡}}=g_{R}-g_{P}$, and a set of coupled harmonic vibrational modes, Q, for the degrees of freedom perpendicular to the reaction coordinate. The origin of this coordinate system is taken to be the MECP itself. In many molecular systems, there are also $f_{0}$ modes with zero frequencies corresponding to translations and rotations, which will be designated by X. The most common case is a nonlinear molecule of $N_{\text{atom}}$ atoms in vacuum, which has $f=3N_{\text{atom}}$ degrees of freedom in total, of which $f_{0}=6$ are zero modes. Three of the X coordinates thus determine the location of the molecular centre of mass, and three of them are Euler angles mass-weighted according to the moments of inertia. Here, we will neglect Coriolis terms [71] and keep the moments of inertia constant using their values at the MECP.

The diabatic potentials are thus approximated by

2.14 $$V_{n}(q,Q)\approx V_{n}^{\text{rxn}}(q)+\frac{1}{2}Q^{T}{\tilde{H}}_{n}Q,$$

where ${\tilde{H}}_{n}=D^{T}H_{n}D$ and D is a $f\times (f-f_{0}-1)$ matrix which projects out the reaction coordinate and $f_{0}$ zero modes corresponding to translations and rotations [70,72]. In general, the two curves $V_{n}^{\text{rxn}}(q)$ can take an arbitrary form as long as they cross. In the simplest case, the potential along the reaction coordinate is assumed to be linear: $V_{n}^{\text{lin}}(q)=V^{\text{‡}}+\kappa_{n}q$, where $\kappa_{n}=g_{n}\cdot g^{\text{‡}}/||g^{\text{‡}}||$ is the slope of the PES at the MECP. One level higher is the harmonic approximation, $V_{n}^{\text{harm}}(q)=V^{\text{‡}}+\kappa_{n}q+\frac{1}{2}m\omega^{2}q^{2}=U_{n}+\frac{1}{2}m\omega^{2}{\left(q-q_{n}\right)}^{2}$, where $q_{n}=-\kappa_{n}/m\omega^{2}$ and $U_{n}=V^{\text{‡}}-\kappa_{n}^{2}/2m\omega^{2}$.

Given the form of equation (2.14), the action is separable, and the parts corresponding to the zero modes and harmonic vibrational modes can be written as analytic functions [6]:

2.15 $$S_{n}(q^{\prime \prime },q^{\prime },X_{-},Q_{-},Q_{+},\tau_{n})=S_{n}^{\text{rxn}}(q^{\prime \prime },q^{\prime },\tau_{n})+\frac{m}{2\tau_{n}}X_{-}^{T}X_{-}+\frac{1}{4}Q_{-}^{T}A_{n}Q_{-}+Q_{+}^{T}B_{n}Q_{+},$$

where $A_{n}=m\Omega_{n}\coth\frac{1}{2}\Omega_{n}\tau_{n}$, $B_{n}=m\Omega_{n}\tanh\frac{1}{2}\Omega_{n}\tau_{n}$ and the matrices $\Omega_{n}$ are defined by $m\Omega_{n}^{2}={\tilde{H}}_{n}$. We have employed the orthogonal coordinate transform $Q_{-}=Q^{\prime }-Q^{\prime \prime }$ and $Q_{+}=\frac{1}{2}(Q^{\prime }+Q^{\prime \prime })$ and likewise for X coordinates (and later also for q).

The action along the reaction coordinate, $S_{n}^{\text{rxn}}$ is defined analogously to equation (2.9). In the case of a linear approximation, $S_{n}^{\text{lin}}=V^{\text{‡}}\tau_{n}+mq_{-}^{2}/2\tau_{n}+\kappa_{n}q_{+}\tau_{n}-\kappa_{n}^{2}\tau_{n}^{3}/24m$, and for the harmonic approximation, $S_{n}^{\text{harm}}=U_{n}\tau_{n}+\frac{1}{4}m\omega q_{-}^{2}\coth\frac{1}{2}\omega \tau_{n}+m\omega {\left(q_{+}-q_{n}\right)}^{2}\tanh\frac{1}{2}\omega \tau_{n}$. However, in general, the action can be evaluated using a Legendre transform

2.16*a* $$\begin{array}{r} S_{n}^{\text{rxn}}(q^{\prime \prime },q^{\prime },\tau_{n})=\mathrm{sgn}(\tau_{n})W_{n}(q^{\prime \prime },q^{\prime },E_{n})+E_{n}\tau_{n} \end{array}$$

2.16*b* $$\begin{array}{r} E_{n}=\frac{\partial S_{n}^{\text{rxn}}}{\partial \tau_{n}}\;\text{and}\;|\tau_{n}|=-\frac{\partial W_{n}}{\partial E_{n}} \end{array}$$

2.16*c* $$\begin{array}{r} \;\mathrm{and}\;W_{n}(q^{\prime \prime },q^{\prime },E_{n})=\int_{q^{\prime }}^{q^{\prime \prime }}|p_{n}\;dq|,\;p_{n}=\sqrt{2m(V_{n}^{\text{rxn}}-E_{n})}, \end{array}$$

where the line integral in equation (2.16c) is along the path from one end point to the turning point and back to the other end point. The sign function, $\mathrm{sgn}(\tau_{n})$, is necessary for treating the inverted regime where $\tau_{P}<0$ [42]. It is thus possible to evaluate all quantities by one-dimensional numerical integration. The total action along the reaction coordinate only is $S_{\text{rxn}}^{\left(2\right)}(q^{\prime },q^{\prime \prime },\tau )=S_{R}^{\text{rxn}}(q^{\prime },q^{\prime \prime },\beta \hbar -\tau )+S_{P}^{\text{rxn}}(q^{\prime \prime },q^{\prime },\tau )$, which is the obvious generalization of the total action of §2(b).

The stationary point is clearly located where $X_{-}=0$ and $Q_{-}=Q_{+}=0$. The remaining variables $q^{\prime }$, $q^{\prime \prime }$ and τ are determined by considering the one-dimensional problem along the reaction coordinate only. At the stationary point, we must have $\partial S^{\left(2\right)}/\partial \tau {|}_{X_{-}=0,Q_{-}=Q_{+}=0}=-\partial S_{R}^{\text{rxn}}/\partial \tau_{R}+\partial S_{P}^{\text{rxn}}/\partial \tau_{P}\equiv -E_{R}+E_{P}=0$ as well as $\partial S^{\left(2\right)}/\partial q^{\prime }=\partial S_{R}^{\text{rxn}}/\partial q^{\prime }+\partial S_{P}^{\text{rxn}}/\partial q^{\prime }\equiv p_{R}^{\prime }-p_{P}^{\prime }=0$ and likewise $p_{R}^{\prime \prime }-p_{P}^{\prime \prime }=0$. These equations enforce energy and momentum conservation and can be solved only at the crossing point, implying that $q^{\prime }=q^{\prime \prime }=0$.

The prefactor terms are given by [18,63]

2.17*a* $$\begin{array}{r} C_{n}=C_{n}^{\text{rxn}}C_{n}^{\text{zero}}C_{n}^{\text{vib}} \end{array}$$

2.17*b* $$\begin{array}{r} C_{n}^{\text{rxn}}=-\frac{\partial^{2}S_{n}^{\text{rxn}}}{\partial q^{\prime }\partial q^{\prime \prime }}=\frac{m^{2}}{p_{n}^{\prime }p_{n}^{\prime \prime }}{\left(\frac{\partial^{2}W_{n}}{\partial {E_{n}}^{2}}\right)}^{-1} \end{array}$$

2.17*c* $$\begin{array}{r} C_{n}^{\text{zero}}={\left(\frac{m}{\tau_{n}}\right)}^{f_{0}} \end{array}$$

2.17*d* $$\begin{array}{r} \;\mathrm{and}\;C_{n}^{\text{vib}}=|m\Omega_{n}\mathrm{csch}\Omega_{n}\tau_{n}|. \end{array}$$

Because in many cases mixed derivatives of position and time vanish at the stationary point, the second-derivative matrix separates into blocks such that the determinant $\Sigma^{\left(2\right)}=\Sigma_{\text{rxn}}^{\left(2\right)}C^{\text{‡}}$, where $\Sigma_{\text{rxn}}^{\left(2\right)}$ has the form of equation (2.13) for the action $S_{\text{rxn}}^{\left(2\right)}(q^{\prime },q^{\prime \prime },\tau )$ and

2.18*a* $$\begin{array}{r} C^{\text{‡}}=C^{\text{‡},\text{zero}}C^{\text{‡},\text{vib}} \end{array}$$

2.18*b* $$\begin{array}{r} C^{\text{‡},\text{zero}}=\left|\frac{\partial^{2}S^{\left(2\right)}}{\partial X_{-}\partial X_{-}}\right|={\left(\frac{m}{\tau_{R}}+\frac{m}{\tau_{P}}\right)}^{f_{0}}={\left(\frac{m\beta \hbar }{\tau_{R}\tau_{P}}\right)}^{f_{0}} \end{array}$$

2.18*c* $$\begin{array}{r} \;\mathrm{and}\;C^{\text{‡},\text{vib}}=|A_{R}+A_{P}||B_{R}+B_{P}|. \end{array}$$

In general, $\Omega_{R}$ and $\Omega_{P}$ have different normal modes and cannot therefore be simultaneously diagonalized. It is therefore not possible to simplify the expression further. Nonetheless, they can at this point be easily evaluated given the Hessians of each state.

The contribution from the zero modes is $\sqrt{C_{R}^{\text{zero}}C_{P}^{\text{zero}}/{\left(2\pi \hbar \right)}^{f_{0}}C^{\text{‡},\text{zero}}}={\left[m/(2\pi \beta \hbar^{2})\right]}^{f_{0}/2}$, which is equivalent to the standard prefactor of a classical configurational integral. Thus, although integration over the zero modes $X_{+}$ cannot be performed by steepest descent, it can be substituted by the classical partition functions instead. This gives a rate (within the separable approximation)

2.19 $$k_{\text{SCI}}^{\left(2\right)}=\sqrt{2\pi \hbar }\;\frac{|\Delta_{\mathrm{RP}}{|}^{2}}{\hbar^{2}}\sqrt{\frac{C_{R}^{\text{rxn}}C_{P}^{\text{rxn}}}{-\Sigma_{\text{rxn}}^{\left(2\right)}}}\frac{Z^{\text{‡}}}{Z_{R}}\;e^{-S_{\text{rxn}}^{\left(2\right)}/\hbar },$$

where the transition-state partition function is $Z^{\text{‡}}=Z^{\text{‡},\text{trans}}Z^{\text{‡},\text{rot}}Z^{\text{‡},\text{vib}}$. Here, the translational and rotational partition functions are given by the usual expressions and are evaluated at the MECP. The reactant partition function, $Z_{R}$, takes the standard form within the (classical) rigid-rotor and (quantum) harmonic-oscillator approximations and takes account of whether a bimolecular or unimolecular reaction is studied. The vibrational partition function of the MECP is given by

2.20 $$Z^{\text{‡},\text{vib}}=\sqrt{\frac{C_{R}^{\text{vib}}C_{P}^{\text{vib}}}{C^{\text{‡},\text{vib}}}},$$

and will be discussed later in this section.

First, we study the effect of tunnelling on the rate within the linear approximation, for which the stationary point is found at $q^{\prime }=q^{\prime \prime }=0$ and $\tau =\beta \hbar \kappa_{R}/(\kappa_{R}-\kappa_{P})$ leading to

2.21 $$\frac{S_{\text{lin}}^{\left(2\right)}}{\hbar }=\beta V^{\text{‡}}-\frac{1}{12}{\left(\frac{\beta }{\beta_{0}}\right)}^{3},$$

where we have defined

2.22 $$\beta_{0}^{3}=\frac{2m}{\hbar^{2}}{\left(\frac{1}{\kappa_{R}}-\frac{1}{\kappa_{P}}\right)}^{2},$$

and the prefactors are $C_{n}^{\text{lin}}=m/\tau_{n}$ and $\Sigma_{\text{lin}}^{\left(2\right)}=-2m(1/\tau_{R}+1/\tau_{P})\frac{1}{2}{\left(\kappa_{P}-\kappa_{R}\right)}^{2}$.

In the classical $\beta \ll \beta_{0}$ limit, we obtain the non-tunnelling harmonic transition-state theory (hTST) rate [12,68]

2.23 $$k_{\text{hTST}}^{\left(2\right)}=\sqrt{\frac{2\pi m}{\beta \hbar^{2}}}\frac{|\Delta_{\mathrm{RP}}{|}^{2}}{\hbar |\kappa_{R}-\kappa_{P}|}\frac{Z^{\text{‡}}}{Z_{R}}\;e^{-\beta V^{\text{‡}}},$$

which is similar in spirit to Eyring’s TST but applicable in the golden-rule limit.

By including the tunnelling effect, the rate is effectively multiplied by a Gamow-like factor:

2.24 $$k_{\text{lin}}^{\left(2\right)}=k_{\text{hTST}}^{\left(2\right)}\;e^{{\left(\beta /\beta_{0}\right)}^{3}/12}.$$

Although we derived this formula using the instanton method, it also happens to be the exact quantum solution for the linear model [41]. The linear model predicts tunnelling at an energy of $E_{\text{lin}}=(1/\hbar )\;\partial S_{\text{lin}}^{\left(2\right)}/\partial \beta =V^{\text{‡}}-\beta^{2}/4\beta_{0}^{3}$. This approach clearly breaks down if $E_{\text{lin}}<\min_{q}V_{R}^{\text{rxn}}(q)$ as this would correspond to an unphysical initial state. In this case, a more appropriate model is definitely required for $V_{n}^{\text{rxn}}(q)$.

It is, however, not much more complicated to employ the separable harmonic approximation, which leads to [12,41]

2.25 $$S_{\text{harm}}^{\left(2\right)}(\tau )=\beta \hbar U_{R}-\varepsilon \tau +4m\omega \xi^{2}\frac{\sinh\frac{1}{2}\omega \tau \sinh\frac{1}{2}(\beta \hbar -\tau )\omega }{\sinh\frac{1}{2}\beta \hbar \omega },$$

where $\varepsilon =U_{R}-U_{P}=(\kappa_{P}^{2}-\kappa_{R}^{2})/2m\omega^{2}$ and $\xi =(\kappa_{R}-\kappa_{P})/2m\omega^{2}$. It has a maximum at $\tau =\frac{1}{2}\beta \hbar -\omega^{-1}\sinh^{-1}[(\varepsilon /2m\omega^{2}\xi^{2})\sinh\frac{1}{2}\beta \hbar \omega ]$. From this, we obtain an instanton rate of

2.26 $$k_{\text{SCI}}^{\left(2\right)}=k_{\text{hTST}}^{\left(2\right)}{\left(\frac{\beta \hbar \omega }{2\sinh\frac{1}{2}\beta \hbar \omega }\right)}^{\frac{1}{2}}{\left(1+\frac{{\left(\kappa_{R}+\kappa_{P}\right)}^{2}}{{\left(\kappa_{R}-\kappa_{P}\right)}^{2}}\sinh^{2}\frac{1}{2}\beta \hbar \omega \right)}^{-\frac{1}{4}}\;e^{\beta V^{\text{‡}}-S_{\text{harm}}^{\left(2\right)}/\hbar }.$$

**Figure 1.**
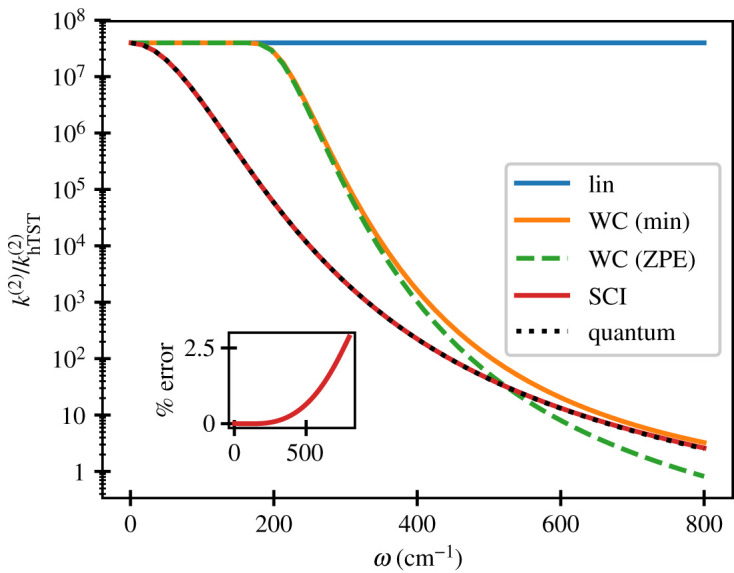
Golden-rule rates obtained for the separable harmonic system using the linear (equation (2.24)) or instanton (SCI) approximation (equation (2.26)) are compared with quantum-mechanical results. Two versions of the WC approximation (equation (2.27)) are also presented which differ in the value of $E_{\text{min}}$ used as indicated. The fixed parameters are $\kappa_{R}/\sqrt{m}=-0.0003\;a.u.$, $\kappa_{P}/\sqrt{m}=-0.0006\;a.u.$ at 300 K. The quantum-mechanical golden-rule result (equation (2.5)) is defined by integrating the correlation function until the first plateau. The inset shows the percentage error made by the instanton approximation over the same range of ω. (Online version in colour.)

The tunnelling factor thus differs considerably from that used in the linear approximation. We show in figure 1 that using the linear approximation can lead to predictions for the tunnelling effect which are orders of magnitude too large if the system deviates from the linear form. The harmonic model tested uses reasonable values for the gradients of the slopes in the inverted regime on the order of those reported in [70]. Here, we have assumed the same frequency for the reactant and product states, but it could be further generalized to treat asymmetric frequencies at the cost of using more complicated formulae [47,73,74].

We also compare with another commonly used formula, the so-called weak-coupling (WC) approximation, which is defined by [70,72,75]

2.27 $$k_{\text{WC}}^{\left(2\right)}=\frac{1}{2\pi \hbar }\frac{Z^{\text{‡}}}{Z_{R}}\int_{E_{\text{min}}}^{\infty }P_{\text{lin}}^{\left(2\right)}(E)\;e^{-\beta E}\;dE,$$

where the transmission probability is given in terms of the Airy function as [75,76]

2.28 $$P_{\text{lin}}^{\left(2\right)}(E)=4\pi^{2}\sqrt{2m\beta_{0}}\;\frac{|\Delta_{\mathrm{RP}}{|}^{2}}{\hbar |\kappa_{R}-\kappa_{P}|}{\mathrm{Ai}}^{2}(\beta_{0}(V^{\text{‡}}-E)),$$

and $\beta_{0}$ was defined in equation (2.22). Two suggestions have appeared in the literature [77], either to choose $E_{\text{min}}$ as the minimum potential energy of the reactants, or the zero-point energy of the reactants. Setting $E_{\text{min}}\to -\infty$ exactly recovers the expression in equation (2.24). The WC approximation is valid when the integrand is dominated by energies near the crossing point, but there is no formal justification for the approach in the deep tunnelling regime, regardless of how $E_{\text{min}}$ is defined, hence the deviation from exact results observed in figure 1.

We now return to the discussion of the vibrational partition function. The formula for $Z^{\text{‡},\text{vib}}$ in equation (2.20) differs from that typically used in the literature. Probably, the most common approach is to quantize the vibrations of an effective Hessian defined by ${\tilde{H}}_{\text{eff}}=\left(\kappa_{P}{\tilde{H}}_{R}-\kappa_{R}{\tilde{H}}_{P})/(\kappa_{P}-\kappa_{R}\right)$ [70,72].

The two methods are in agreement in the classical limit as we can prove by expanding the trigonometric functions to first order in their arguments to obtain $Z^{\text{‡},\text{vib}}\approx |\beta \hbar \Omega_{\text{eff}}{|}^{-1}$ where $\Omega_{\text{eff}}^{2}=(\Omega_{R}^{2}\tau_{R}+\Omega_{P}^{2}\tau_{P})/\beta \hbar$. If we take the solution of τ from the linear approximation (which is also valid at high temperature for an arbitrary system) [41], we recover $m\Omega_{\text{eff}}^{2}={\tilde{H}}_{\text{eff}}$.

However, at low temperatures, the commonly used approximation [70],

2.29 $$Z^{\text{‡},\text{vib}}\approx |2\sinh\frac{1}{2}\beta \hbar \Omega_{\text{eff}}{|}^{-1},$$

can only be derived from equation (2.20) in the limiting case that $\Omega_{R}-\Omega_{P}\to 0$. In conclusion, the usual formula (equation (2.29)) is only rigorously justified in the classical limit or in the case that the difference between the two Hessians is negligible. However, for consistency with the separable assumption made in equation (2.14), we recommend that one should use the value of τ determined from the stationary point and the general formula, equation (2.20).

## 3. Ring-polymer formulation

In this section, we present the working equations for the ring-polymer formulation of the golden-rule instanton. In particular, we go beyond previous work [42,46,47] in three aspects: we present an optimization algorithm using only half of the ring polymer; we obtain the rate directly from the eigenvalues of the ring-polymer Hessian; and we explicitly treat translational and rotational degrees of freedom. This makes the algorithm as similar as possible to the standard ring-polymer instanton method within the Born–Oppenheimer approximation [18].

### (a) Ring-polymer rate

As described in [42,46], we discretize the instanton path and its associated action in the form of a ring polymer. First, the instanton is located using a saddle-point search in the extended ring-polymer space as described in §3(b). Then, instead of evaluating the rate via equation (2.12), which is defined in terms of continuous trajectories and van-Vleck propagators, it is more natural to obtain the result directly from the ring-polymer picture. This approach is in direct analogy to the standard Born–Oppenheimer ring-polymer instanton method [17–20,78,79].

The ring-polymer rate expression can be readily derived by applying the Trotter formula to split each of the propagators $K_{n}$ in equation (2.7) into $N_{n}$ intervals of imaginary time, $\epsilon_{n}=\tau_{n}/N_{n}$, and inserting a complete set of position states between each interval before applying the steepest-descent approximation. We thus discretize the instanton orbit into $N=N_{R}+N_{P}$ ‘beads’ which constitute replicas of the system along the pathway. In order to draw a direct connection to the application of the method to molecular systems, we introduce $r_{i,a}$ as the three-dimensional atomic coordinate vector for atom a of bead i with corresponding mass $m_{a}$. The atomic vectors for bead i can be combined into the f-dimensional coordinate vector $r_{i}$, where $f=3N_{\text{atom}}$ and $N_{\text{atom}}$ is the number of atoms. This results in the following expression for the correlation function at t=0:^2^

3.1 $$c^{\left(2\right)}(\tau )=\prod_{a=1}^{N_{\text{atom}}}{\left(\frac{m_{a}}{2\pi \hbar \epsilon_{R}}\right)}^{3N_{R}/2}{\left(\frac{m_{a}}{2\pi \hbar \epsilon_{P}}\right)}^{3N_{P}/2}\int \Delta_{\text{RP}}({\text{r}}_{N_{R}})\Delta_{\text{RP}}^{*}({\text{r}}_{N})\;e^{-S_{N}^{\left(2\right)}/\hbar }\;dr,$$

where $r=\{r_{1},\ldots ,r_{N}\}$ is the combined vector of all bead positions, and the action is

3.2 $$\begin{array}{rl} S_{N}^{\left(2\right)}\equiv S_{N}^{\left(2\right)}(r,\tau ) & \;=\sum_{i=1}^{N_{R}}\left(\sum_{a=1}^{N_{\text{atom}}}\frac{m_{a}||r_{i,a}-r_{i-1,a}|{|}^{2}}{2\epsilon_{R}}+\frac{1}{2}\epsilon_{R}[V_{R}({\text{r}}_{i})+V_{R}({\text{r}}_{i-1})]\right)\\ & \;\;+\sum_{i=N_{R}+1}^{N}\left(\sum_{a=1}^{N_{\text{atom}}}\frac{m_{a}||r_{i,a}-r_{i-1,a}|{|}^{2}}{2\epsilon_{P}}+\frac{1}{2}\epsilon_{P}[V_{P}({\text{r}}_{i})+V_{P}({\text{r}}_{i-1})]\right), \end{array}$$

where cyclic indices are implied such that $r_{0}\equiv r_{N}$, forming a closed ring. Equation (3.2) is defined such that in the $N_{R},N_{P}\to \infty$ limit the value of the ring-polymer action at its stationary point is equal to the SCI action, $S^{\left(2\right)}$. Equivalently to the derivation in §2(b), we seek to obtain the instanton rate expression by simultaneous steepest-descent integration about the stationary point of $S_{N}^{\left(2\right)}$ in r and τ. However, before this can be done, we must account for the zero-frequency modes corresponding to global translations and rotations of the ring polymer. Note that, in contrast to the standard Born–Oppenheimer instanton, there is no zero mode corresponding to permutations of the beads here.

In analogy to [18], we will write the ring-polymer instanton rate as a product of translational, rotational and vibrational contributions

3.3 $$k_{\text{SCI}}^{\left(2\right)}Z_{R}=\sqrt{2\pi }\;\bar{\tau }\;\frac{|\Delta_{\mathrm{RP}}{|}^{2}}{\hbar^{2}}Z_{\text{trans}}^{\text{‡}}Z_{\text{rot}}^{\text{‡}}Z_{\text{vib}}^{\text{‡}}\;e^{-\bar{\epsilon }\;U_{N}^{\left(2\right)}/\hbar },$$

where $\bar{\tau }=\tau_{R}+|\tau_{P}|$, which is equivalent to βℏ in the normal but not in the inverted regime (where $\tau_{P}<0$), $\bar{\epsilon }=\bar{\tau }/N$ and $U_{N}^{\left(2\right)}=S_{N}^{\left(2\right)}/\;\bar{\epsilon }$ is the ring-polymer potential of the instanton configuration. The number of beads on the reactant and product states can be chosen arbitrarily and independently of each other, which gives rise to an unappealing feature. Namely, even though the total rate is independent of the ratio $N_{R}:N_{P}$ as long as the instanton is converged with respect $N_{R}$ and $N_{P}$, the values of the rotational and vibrational partition functions can change depending on the user’s choice for the bead split when the standard definitions for the ring-polymer partition functions are employed. We can resolve this issue by adopting a bead-dependent mass-weighting scheme that renormalizes the individual partition functions and renders them independent of the chosen bead distribution.

We define the bead-dependent masses

3.4*a* $$\begin{array}{r} m_{i,a}=\frac{m_{a}\epsilon_{R}}{\bar{\epsilon }}\;\forall i\in \{1,\ldots ,N_{R}-1\}, \end{array}$$

3.4*b* $$\begin{array}{r} m_{i,a}=\frac{m_{a}|\epsilon_{P}|}{\bar{\epsilon }}\;\forall i\in \{N_{R}+1,\ldots ,N-1\}, \end{array}$$

3.4*c* $$\begin{array}{r} \;\mathrm{and}\;m_{N_{R},a}=m_{N,a}=\frac{m_{a}(\epsilon_{R}+|\epsilon_{P}|)}{2\bar{\epsilon }}. \end{array}$$

This mass-weighting scheme does not alter the standard ring-polymer expression for $Z_{\text{trans}}^{\text{‡}}$, as the total mass of the ring polymer remains equal to NM, where $M=\sum_{a=1}^{N_{\text{atom}}}m_{a}$ is the total mass of the molecule. The moment-of-inertia tensor for the ring polymer, however, is affected by the mass-weighting

3.5 $${\text{I}}_{N}=\sum_{i=1}^{N}\sum_{a=1}^{N_{\text{atom}}}m_{i,a}[(r_{i,a}\cdot r_{i,a})I-(r_{i,a}⊗r_{i,a})],$$

where I is the 3×3 identity matrix, ⊗ is the outer product and we have assumed that the centre of mass has been placed at the origin, i.e. $\sum_{i=1}^{N}\sum_{a=1}^{N_{\text{atom}}}m_{i,a}r_{i,a}=0$. Note that in equation (3.5) the mass $m_{i,a}$ is not only different for different atoms but also for different beads, in line with our definition in equations (3.4). Given $I_{N}$, the rotational partition function is then defined by the standard equations for linear or nonlinear configurations (e.g. eqn (60) in [18]).

The vibrational partition function is defined by

3.6 $$Z_{\text{vib}}^{\text{‡}}=N^{f_{0}}\Xi_{N}\prod_{k=f_{0}+1}^{Nf+1}\left|\frac{1}{\bar{\epsilon }\eta_{k}}\right|,$$

where $\eta_{k}^{2}$ are the Nf+1 eigenvalues of the mass-weighted ring-polymer Hessian defined by $M^{-\frac{1}{2}}[\nabla_{r,\tau }^{2}U_{N}^{\left(2\right)}]M^{-\frac{1}{2}}$, with the gradient with respect to bead positions and imaginary time combined $\nabla_{r,\tau }=[\partial /\partial r_{1},\ldots ,\partial /\partial r_{N},\partial /\partial \tau$] and the mass matrix $M=\text{diag}(m_{1},\ldots ,m_{N},\zeta_{\tau })$ containing the bead-dependent masses defined in equations (3.4). In analogy to the coordinate vector $r_{i}$ of bead i, we defined the corresponding mass vectors $m_{i}$ containing the masses associated with all f degrees of freedom. Here, we also choose to weight the imaginary-time coordinate by $\zeta_{\tau }=\hbar /\bar{\tau }N$ to ensure all entries of the ring-polymer Hessian have the same unit. The $f_{0}$ zero eigenvalues corresponding to global translations and rotations are excluded from the product in equation (3.6). The eigenvalues associated with the single imaginary mode in the normal regime, or the $N_{P}f+1$ imaginary modes occurring in the inverted regime (see appendix A and [42]), enter the product with their absolute magnitude. Finally, there is a factor $\Xi_{N}$, which accounts for the masses and imaginary-time intervals in the prefactor from equation (3.1), as well as the mass-weighting. It is given by

3.7 $$\Xi_{N}=\sqrt{\frac{\hbar }{N\bar{\tau }\zeta_{\tau }}}{\left(\frac{{\bar{\epsilon }}^{N}}{{\epsilon_{R}}^{N_{R}}|\epsilon_{P}{|}^{N_{P}}}\right)}^{f/2}\prod_{i=1}^{N}\prod_{a=1}^{N_{\text{atom}}}{\left(\frac{m_{a}}{m_{i,a}}\right)}^{3/2}.$$

Note that this factor is equal to 1 for the mass-weighting scheme introduced above if the bead ratios are chosen such that $\epsilon_{R}=|\epsilon_{P}|$. We emphasize that different choices of mass-weighting schemes if implemented correctly (i.e. taking the factor $\Xi_{N}$ into account) do not affect the total rate, but only the relative values of the vibrational, rotational and translational contributions. For a molecule with no translational or rotational degrees of freedom, the ring-polymer instanton rate (equation (3.3)) reduces to equation (2.12) as the number of beads tends to infinity.

### (b) Optimization algorithms

In this subsection, we will discuss various numerically efficient algorithms that can be used to locate golden-rule instantons within the ring-polymer formalism. As has been mentioned earlier, this is equivalent to finding a saddle point of the ring-polymer action (equation (3.2)). In the normal regime, as for the Born–Oppenheimer instanton, this saddle point is of index one, i.e. the second-derivative matrix of the action exhibits one negative eigenvalue, and thus the standard transition-state optimization routines can be applied, typically quasi-Newton algorithms [80–82]. The key difference is that we must also simultaneously optimize τ along with r.

**Figure 2.**
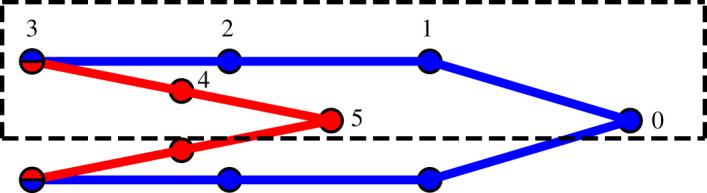
Illustration of how a half ring polymer consisting of six beads (dashed box) relates to the corresponding 10-bead full ring polymer. Here, $N_{R}=6$ and $N_{P}=4$. Note that the configuration of beads shown here is typical for an instanton in the inverted regime, although the numbering scheme is identical in the normal regime. (Online version in colour.)

Analogously to the Born–Oppenheimer instanton [18,20,83], the trajectories of the golden-rule instanton typically fold back on themselves [46]. In these cases, we can approximately halve the computational effort required for evaluating the action and its derivatives. We may therefore locate the instanton by searching for a single-index saddle point of the half ring-polymer action,

3.8 $$\begin{array}{rl} S_{N/2}^{\left(2\right)}\equiv S_{N/2}^{\left(2\right)}(r,\tau ) & \;=\sum_{i=1}^{N_{R}/2}\left(\sum_{a=1}^{N_{\text{atom}}}\frac{m_{a}||r_{i,a}-r_{i-1,a}|{|}^{2}}{2\epsilon_{R}}+\frac{1}{2}\epsilon_{R}[V_{R}({\text{r}}_{i})+V_{R}({\text{r}}_{i-1})]\right)\\ & \;\;+\sum_{i=N_{R}/2+1}^{N/2}\left(\sum_{a=1}^{N_{\text{atom}}}\frac{m_{a}||r_{i,a}-r_{i-1,a}|{|}^{2}}{2\epsilon_{P}}+\frac{1}{2}\epsilon_{P}[V_{P}({\text{r}}_{i})+V_{P}({\text{r}}_{i-1})]\right), \end{array}$$

which is defined such that $2S_{N/2}^{\left(2\right)}=S_{N}^{\left(2\right)}$ for the instanton configuration and $r=\{r_{0},\ldots ,r_{N/2}\}$ has been redefined for the half ring polymer. Note that the bead labelling has changed compared with equation (3.2), since cyclic indexing does not apply to the half ring polymer and there is only a single hopping bead (figure 2).

When optimizing an instanton for a new system, one can use the fact that at high temperatures the instanton will be compact and located near the MECP, which can itself be found with standard methods [84–90]. A good initial guess for the value of τ is given by the classical, short-time limit of the action (see discussion above equation (2.21)) [41]. It is therefore relatively easy to optimize an instanton at high temperature, which can then serve as a starting point to slowly cool down the system. This process is accompanied by the gradual expansion of the instanton.

In contrast to the normal regime, in the inverted regime of an exoergic reaction, the ring-polymer instanton corresponds to a saddle point of index $N_{P}f+1$.^3^ In the inverted regime, we can also employ the half ring-polymer approach (equation (3.8)), though here the index of the required saddle point changes to $N_{P}f/2+1$. From figure 2, it can be seen how the half ring polymer emerges from the full ring in a configuration typically assumed in the inverted regime.

Due to the fact that in the inverted regime one is no longer seeking a single-index saddle point, different optimization algorithms are required. Conveniently, we can make use of algorithms originally developed for locating higher-index saddle points in Lennard–Jones clusters [91–93]. These algorithms are based on eigenvector following, where uphill steps are taken in the direction of the eigenvectors corresponding to the lowest $N_{P}f/2+1$ eigenvalues and downhill steps are taken in the remaining degrees of freedom. Hence, even if the initial guess does not have the required number of negative eigenvalues, the eigenvector-following algorithm will guide us towards the stationary point of the correct index. In principle, it is also possible to employ standard Newton–Raphson optimizers. These, however, require an initial guess with the precisely right index, since the algorithm cannot change the signs of eigenvalues.

As in conventional geometry optimization, it is often helpful to project out global rotations and translations. This can be done with standard methods [93], treating the ring polymer as a ‘supermolecule’ [18]. It is however necessary to extend the projection matrices into the combined {r,τ} space by appending zeros for the extra τ degree of freedom.

### (c) Application to molecular dissociation

Consider the rate of unimolecular dissociation of a dimer AB defined by the intramolecular pair potentials

3.9 $$V_{R}(r)=D_{R}{\left(1-e^{-\alpha (r-r_{\text{e}})}\right)}^{2}\;\mathrm{and}\;V_{P}(r)=D_{P}\;e^{-2\alpha (r-r_{\text{e}})}-\varepsilon_{P},$$

where $r=||r_{\text{A}}-r_{\text{B}}||$ is the bond length. We chose physically reasonable parameters similar to those of [94] (although the process studied in that paper is subtly different): $D_{R}=5\;\text{eV}$, $D_{P}=3.2\;\text{eV}$, $\alpha =1.96\;{\text{Å}}^{-1}$, $r_{\text{e}}=1\;\text{Å}$, $\varepsilon_{P}=1.2\;\text{eV}$ and a reduced mass of $\mu =m_{\text{A}}m_{\text{B}}/(m_{\text{A}}+m_{\text{B}})=1\;\text{u}$. The dimer is initialized in a thermal equilibrium of the bound metastable state |R⟩ and decays via electron transfer into the |P⟩ state, from where it can dissociate.

**Figure 3.**
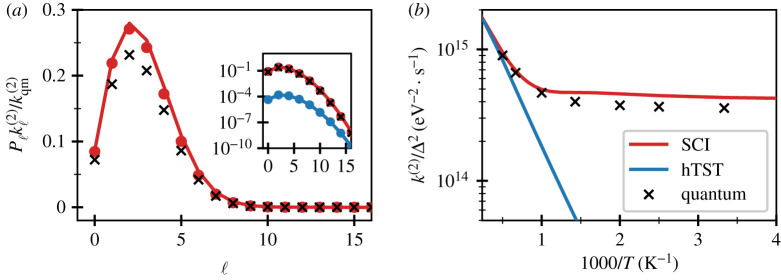
Results for the rate of dissociation of the model dimer system defined in equations (3.9). (*a*) Contributions, $P_{\ell }k_{\ell }^{\left(2\right)}$, to the thermal rate at T=300 K from different values of angular momentum quantum number, ℓ, where $P_{\ell }=(2\ell +1)Z_{\ell }/Z_{R}$. The dots are evaluated using the semiclassical instanton (SCI) approximation based on an effective one-dimensional system including the centrifugal potential, whereas the lines of the same colour correspond to a rigid-rotor approximation. Results are normalized by the total quantum-mechanical rate $k_{\text{qm}}^{\left(2\right)}$. The inset shows the same plot on a logarithmic scale and in addition includes harmonic transition-state theory (hTST) results. (*b*) Thermal reaction rates computed with SCI, hTST and quantum mechanics. (Online version in colour.)

Quantum-mechanical results were obtained from Fermi’s golden rule by numerically solving the effective one-dimensional Schrödinger equations using quadrature on a polynomial basis for the bound potential and Numerov’s method for the unbound state. In each case, a centrifugal term B(r)ℓ(ℓ+1) was added to the potential, where $B(r)=\hbar^{2}/2\mu r^{2}$ [95,96], to give a rate $k_{\ell }^{\left(2\right)}$ thermally averaged over vibrational modes for the given angular momentum quantum number and associated partition function $Z_{\ell }$. Finally, rates were summed over the angular momentum quantum number, ℓ, and weighted by the degeneracy, to give the total thermal rate $k^{\left(2\right)}Z_{R}=\sum_{\ell }(2\ell +1)k_{\ell }^{\left(2\right)}Z_{\ell }$, where $Z_{R}=\sum_{\ell }(2\ell +1)Z_{\ell }$. Figure 3*a* shows the contributions from the various terms evaluated using the exact quantum expressions, as well as via SCI theory and harmonic transition-state theory. The instanton approximation leads to a slight overprediction of the rates, whereas the hTST results are orders of magnitude too small. In both cases, however, it can be seen that for this diatomic there is little deviation between the predictions based on summing over angular momentum quantum numbers, and those based on the rigid-rotor approximation. In polyatomic molecules, the situation is expected to be even more favourable, as the moments of inertia are typically larger and do not change considerably along the tunnelling pathway. Note that the effective rotational constant in the instanton picture is $B_{\text{SCI}}=1.89\;\text{meV}$, whereas for hTST it is given by the value at the MECP, $B_{\text{hTST}}=1.52\;\text{meV}$. As the predicted total thermal rate is inversely proportional to this value, the agreement with the quantum results has been improved by using $B_{\text{SCI}}$ instead of $B_{\text{hTST}}$.

In figure 3*b*, results are presented for the golden-rule rate constant obtained by instanton theory and harmonic TST, which can be compared with a benchmark quantum calculation. As expected, there is a plateau in the Arrhenius plot, which is also observed as a manifestation of low-temperature tunnelling effects within the Born–Oppenheimer paradigm [97]. This can be explained within the quantum-mechanical picture, as only the ground state is occupied at low temperatures. In instanton theory, the tunnelling energy $E_{n}$ tends to zero (using the minimum of $V_{R}$ as zero on the energy scale) as the temperature is lowered, and $S^{\left(2\right)}$ tends to a constant, thus explaining why the rate becomes independent of T. The semiclassical approximations behind instanton theory clearly remain reasonably accurate even in this extreme regime, with the rates overestimated by only 20%. Note that, even though the instanton energy tends towards the bottom of the reactant well, the theory does not neglect the effect of ZPE, which is instead dealt with in the prefactor terms. In fact, the same thing happens in the Born–Oppenheimer case, for which it is possible to prove that the low-temperature limit of instanton theory is asymptotic to the WKB rate of tunnelling out of the lowest vibrational level [97]. Because of this the instanton results are in good agreement with the quantum calculations in the deep-tunnelling regime and correctly recover the classical limit at high temperatures.

## 4. Fourth-order instanton theory

**Figure 4.**
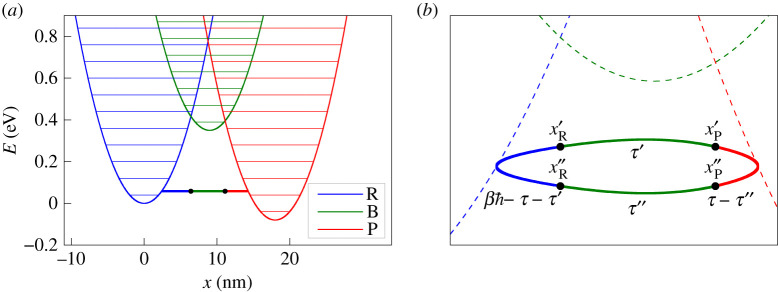
(*a*) A simple model system composed of reactant, bridge and product potential-energy surfaces. The vibrational energy levels of each PES are given by thin horizontal lines. The instanton is shown by a thick horizontal line at the optimal tunnelling energy. (*b*) A schematic illustrating the instanton for an arbitrary set of PESs. The trajectories are shown with bold lines and the PESs with dashed lines, in each case with the colour corresponding to the diabatic state. The label below a trajectory denotes the imaginary time for travelling along it. The hopping points $x_{R}^{\prime }$, $x_{P}^{\prime }$, $x_{R}^{\prime \prime }$ and $x_{P}^{\prime \prime }$ are shown as black dots and labelled above the corresponding point. (Online version in colour.)

We consider a system that consists of three potentials corresponding to a reactant |R⟩, bridge |B⟩ and product |P⟩, arranged as shown in figure 4*a*. In particular, we exclusively consider the case for which the reaction is in the normal regime and the minimum of the bridge is higher in energy than both the reactant and product minima, but at the point where the reactant and product cross (with energy $V_{\text{RP}}=V_{\text{R}}(x)=V_{\text{P}}(x)$), the bridge PES is lowest. We assume that |R⟩ and |P⟩ are coupled to |B⟩ but that |R⟩ is not directly coupled to |P⟩. This may be rigorously a consequence of selection rules based on symmetry, or only an approximation in which it is valid to neglect the direct mechanism due to the higher-energy crossing point. It is clear that for this problem, we require a fourth-order rate expression, such as equation (1.2), which is given by Dirac in his classic textbook [5]. In this section, we will derive an instanton approximation for this rate.

### (a) Derivation of the fourth-order instanton rate

We write the total Hamiltonian as a sum of an uncoupled system and a small perturbation

4.1 $$\hat{H}={\hat{H}}^{\left(0\right)}+{\hat{H}}^{\left(1\right)},$$

with

4.2*a* $$\begin{array}{rl} & {\hat{H}}^{\left(0\right)}={\hat{H}}_{R}|R\rangle \langle R|+{\hat{H}}_{B}|B\rangle \langle B|+{\hat{H}}_{P}|P\rangle \langle P| \end{array}$$

4.2*b* $$\begin{array}{rl} & {\hat{H}}^{\left(1\right)}={\hat{\Delta }}_{\mathrm{RB}}(|R\rangle \langle B|+|B\rangle \langle R|)+{\hat{\Delta }}_{\mathrm{BP}}(|B\rangle \langle P|+|P\rangle \langle B|), \end{array}$$

where ${\hat{H}}_{R}$ and ${\hat{H}}_{P}$ are defined as before and ${\hat{H}}_{B}=\frac{{\hat{p}}^{2}}{2m}+V_{B}(\hat{x})$ is the Hamiltonian corresponding to the bridge PES, $V_{B}(x)$. The derivation that follows uses a similar approach to that of the second-order rate from §2(a). In addition to equation (2.3), we now also have the eigenvalue equation

4.3 $${\hat{H}}_{B}|\chi_{B}^{\lambda }\rangle =E_{B}^{\lambda }|\chi_{B}^{\lambda }\rangle .$$

The system we are studying evolves from an initial vibrational state $|\chi_{R}^{\mu }\rangle$ to a final vibrational state $|\chi_{P}^{\nu }\rangle$ via the intermediate vibrational states $|\chi_{B}^{\lambda }\rangle$. If we average equation (1.2) over a thermal Boltzmann distribution of the reactant states (with partition function $Z_{R}$ as before), the rate expression for this system becomes

4.4 $$\begin{array}{rl} k^{\left(4\right)}Z_{R} & \;=\frac{2\pi }{\hbar }\sum_{\mu \nu }e^{-[(\beta \hbar -\tau )E_{R}^{\mu }+\tau E_{P}^{\nu }]/\hbar }\;\delta (E_{P}^{\nu }-E_{R}^{\mu })\\ & \;\times \sum_{\lambda^{\prime }\lambda^{\prime \prime }}\frac{\left\langle \chi_{R}^{\mu }|{\hat{\Delta }}_{\mathrm{RB}}|\chi_{B}^{\lambda^{\prime }}\rangle \langle \chi_{B}^{\lambda^{\prime }}|{\hat{\Delta }}_{\mathrm{BP}}|\chi_{P}^{\nu }\right\rangle }{E_{B}^{\lambda^{\prime }}-E_{R}^{\mu }}\frac{\left\langle \chi_{P}^{\nu }|{\hat{\Delta }}_{\mathrm{BP}}^{†}|\chi_{B}^{\lambda^{\prime \prime }}\rangle \langle \chi_{B}^{\lambda^{\prime \prime }}|{\hat{\Delta }}_{\mathrm{RB}}^{†}|\chi_{R}^{\mu }\right\rangle }{E_{B}^{\lambda^{\prime \prime }}-E_{P}^{\nu }}. \end{array}$$

Note that we have chosen to write the expression in this symmetric form using the presence of the delta function to freely interchange the energies of the initial, $E_{R}^{\mu }$, and final states, $E_{P}^{\nu }$.

In order to derive an instanton approximation, we write the delta function and the 1/E terms as integrals over real and imaginary time, respectively

4.5*a* $$\delta (E)=\frac{1}{2\pi \hbar }\int_{-\infty }^{\infty }e^{iEt/\hbar }\;dt$$

and

4.5*b* $$\frac{1}{E}=\frac{1}{\hbar }\int_{0}^{\infty }e^{-E\tau^{\prime }/\hbar }\;d\tau^{\prime }.$$

Note that equation (4.5b) is only valid if E>0. For our problem, this means that the bridge states have to be sufficiently high above the minimum of both the reactant and product PESs, such that the Boltzmann factor makes the terms with negative values of $\left(E_{B}^{\lambda^{\prime }}-E_{R}^{\mu }\right)$ and $\left(E_{B}^{\lambda^{\prime \prime }}-E_{P}^{\nu }\right)$ negligible. The following expressions are therefore asymptotic to the exact rate in the limit of high bridge energies.

The physical interpretation of this assumption is that the reaction proceeds via a concerted single-step mechanism from R to P via virtual B states, rather than a sequential two-step process that goes from R to B followed by B to P [49]. In the context of instanton theory, this implies that the instanton should be located at an energy below the minimum of the bridge, i.e. this is a deep-tunnelling process.

Collecting the energy terms, making use of the eigenvalue equations (2.3) and (4.3) and the additional resolution of identity $\sum_{\lambda }|\chi_{B}^{\lambda }\rangle \langle \chi_{B}^{\lambda }|=1$, we find

4.6 $$k^{\left(4\right)}Z_{R}=\frac{1}{\hbar^{4}}\int_{-\infty }^{\infty }dt\int_{0}^{\infty }d\tau^{\prime }\int_{0}^{\infty }d\tau^{\prime \prime }\;c^{\left(4\right)}(\tau +it,\tau^{\prime },\tau^{\prime \prime }),$$

where the three-time correlation function is

4.7 $$\begin{array}{rl} c^{\left(4\right)}(\tau +it,\tau^{\prime },\tau^{\prime \prime }) & \;=\mathrm{Tr}[{\hat{\Delta }}_{\mathrm{RB}}^{†}\;e^{-{\hat{H}}_{R}(\beta \hbar -\tau -it-\tau^{\prime })/\hbar }\;{\hat{\Delta }}_{\mathrm{RB}}\;e^{-{\hat{H}}_{B}\tau^{\prime }/\hbar }\\ & \;\times {\hat{\Delta }}_{\mathrm{BP}}\;e^{-{\hat{H}}_{P}(\tau +it-\tau^{\prime \prime })/\hbar }\;{\hat{\Delta }}_{\mathrm{BP}}^{†}\;e^{-{\hat{H}}_{B}\tau^{\prime \prime }/\hbar }]. \end{array}$$

This correlation function is an extension of the one from the second-order case (equation (2.6)). In between the propagators for |R⟩ and |P⟩, we now have to propagate on the bridge, |B⟩.

Using the same arguments as those for equation (2.5) in §2(a), the overall rate calculated using equation (4.6) is independent of the choice of τ. As in §2(b), we can write this correlation function in terms of imaginary-time propagators

4.8 $$\begin{array}{rl} c^{\left(4\right)} & \;=⨌\Delta_{\mathrm{RB}}^{*}(x_{R}^{\prime \prime })K_{R}(x_{R}^{\prime \prime },x_{R}^{\prime },\beta \hbar -\tau -it-\tau^{\prime })\Delta_{\mathrm{RB}}(x_{R}^{\prime })K_{B}(x_{R}^{\prime },x_{P}^{\prime },\tau^{\prime })\\ & \;\times \Delta_{\mathrm{BP}}(x_{P}^{\prime })K_{P}(x_{P}^{\prime },x_{P}^{\prime \prime },\tau +it-\tau^{\prime \prime })\Delta_{\mathrm{BP}}^{*}(x_{P}^{\prime \prime })K_{B}(x_{P}^{\prime \prime },x_{R}^{\prime \prime },\tau^{\prime \prime })\;dx_{R}^{\prime }\;dx_{P}^{\prime }\;dx_{R}^{\prime \prime }\;dx_{P}^{\prime \prime }. \end{array}$$

Next, we invoke the semiclassical approximation and use kernels that correspond to classical trajectories in imaginary time from equation (2.8). When we perform steepest-descent integration over all variables, the four trajectories combine into a periodic orbit, which we identify as the generalized fourth-order instanton with action

4.9 $$S^{\left(4\right)}=S_{R}(x_{R}^{\prime \prime },x_{R}^{\prime },\beta \hbar -\tau -\tau^{\prime })+S_{B}(x_{R}^{\prime },x_{P}^{\prime },\tau^{\prime })+S_{P}(x_{P}^{\prime },x_{P}^{\prime \prime },\tau -\tau^{\prime \prime })+S_{B}(x_{P}^{\prime \prime },x_{R}^{\prime \prime },\tau^{\prime \prime }),$$

with each component defined according to equation (2.9). The resulting trajectory is shown in figure 4*b*. In complete analogy with the golden-rule case, the stationary-point conditions ensure that we have momentum and energy conservation between each segment of the path, leading to a periodic orbit of imaginary time βℏ.

The instanton approximation to the rate is therefore

4.10 $$k_{\text{SCI}}^{\left(4\right)}Z_{R}={\left(2\pi \hbar \right)}^{3/2}\frac{|\Delta_{\mathrm{RB}}{|}^{2}|\Delta_{\mathrm{BP}}{|}^{2}}{\hbar^{4}}\sqrt{\frac{C_{R}C_{B}^{\prime }C_{P}C_{B}^{\prime \prime }}{-\Sigma^{\left(4\right)}}}\;e^{-S^{\left(4\right)}/\hbar },$$

where $C_{R}$ and $C_{P}$ are defined equivalently to equation (2.10), $C_{B}^{\prime }=\left|-\frac{\partial^{2}S_{B}^{\prime }}{\partial x_{R}^{\prime }\partial x_{P}^{\prime }}\right|$, where $S_{B}^{\prime }\equiv S_{B}(x_{R}^{\prime },x_{P}^{\prime },\tau^{\prime })$, and similarly for $C_{B}^{\prime \prime }$ and $S_{B}^{\prime \prime }$. The determinant of the second derivatives of the total action is

$$\Sigma^{\left(4\right)}=\left|\begin{array}{ccccccc} \frac{\partial^{2}\left(S_{R}+S_{B}^{\prime }\right)}{\partial x_{R}^{\prime }\partial x_{R}^{\prime }} & \frac{\partial^{2}S_{B}^{\prime }}{\partial x_{R}^{\prime }\partial x_{P}^{\prime }} & \frac{\partial^{2}S_{R}}{\partial x_{R}^{\prime }\partial x_{R}^{\prime \prime }} & 0 & \frac{\partial^{2}\left(S_{R}+S_{B}^{\prime }\right)}{\partial x_{R}^{\prime }\partial \tau^{\prime }} & 0 & \frac{\partial^{2}S_{R}}{\partial x_{R}^{\prime }\partial \tau }\\ \frac{\partial^{2}S_{B}^{\prime }}{\partial x_{P}^{\prime }\partial x_{R}^{\prime }} & \frac{\partial^{2}\left(S_{P}+S_{B}^{\prime }\right)}{\partial x_{P}^{\prime }\partial x_{P}^{\prime }} & 0 & \frac{\partial^{2}S_{P}}{\partial x_{P}^{\prime }\partial x_{P}^{\prime \prime }} & \frac{\partial^{2}S_{B}^{\prime }}{\partial x_{P}^{\prime }\partial \tau^{\prime }} & \frac{\partial^{2}S_{P}}{\partial x_{P}^{\prime }\partial \tau^{\prime \prime }} & \frac{\partial^{2}S_{P}}{\partial x_{P}^{\prime }\partial \tau }\\ \frac{\partial^{2}S_{R}}{\partial x_{R}^{\prime \prime }\partial x_{R}^{\prime }} & 0 & \frac{\partial^{2}\left(S_{B}^{\prime \prime }+S_{R}\right)}{\partial x_{R}^{\prime \prime }\partial x_{R}^{\prime \prime }} & \frac{\partial^{2}S_{B}^{\prime \prime }}{\partial x_{R}^{\prime \prime }\partial x_{P}^{\prime \prime }} & \frac{\partial^{2}S_{R}}{\partial x_{R}^{\prime \prime }\partial \tau^{\prime }} & \frac{\partial^{2}S_{B}^{\prime \prime }}{\partial x_{R}^{\prime \prime }\partial \tau^{\prime \prime }} & \frac{\partial^{2}S_{R}}{\partial x_{R}^{\prime \prime }\partial \tau }\\ 0 & \frac{\partial^{2}S_{P}}{\partial x_{P}^{\prime \prime }\partial x_{P}^{\prime }} & \frac{\partial^{2}S_{B}^{\prime \prime }}{\partial x_{P}^{\prime \prime }\partial x_{R}^{\prime \prime }} & \frac{\partial^{2}\left(S_{B}^{\prime \prime }+S_{P}\right)}{\partial x_{P}^{\prime \prime }\partial x_{P}^{\prime \prime }} & 0 & \frac{\partial^{2}\left(S_{B}^{\prime \prime }+S_{P}\right)}{\partial x_{P}^{\prime \prime }\partial \tau^{\prime \prime }} & \frac{\partial^{2}S_{P}}{\partial x_{P}^{\prime \prime }\partial \tau }\\ \frac{\partial^{2}\left(S_{B}^{\prime }+S_{R}\right)}{\partial \tau^{\prime }\partial x_{R}^{\prime }} & \frac{\partial^{2}S_{B}^{\prime }}{\partial \tau^{\prime }\partial x_{P}^{\prime }} & \frac{\partial^{2}S_{R}}{\partial \tau^{\prime }\partial x_{R}^{\prime \prime }} & 0 & \frac{\partial^{2}\left(S_{B}^{\prime }+S_{R}\right)}{\partial {\tau^{\prime }}^{2}} & 0 & \frac{\partial^{2}S_{R}}{\partial \tau^{\prime }\partial \tau }\\ 0 & \frac{\partial^{2}S_{P}}{\partial \tau^{\prime \prime }\partial x_{P}^{\prime }} & \frac{\partial^{2}S_{B}^{\prime \prime }}{\partial \tau^{\prime \prime }\partial x_{R}^{\prime \prime }} & \frac{\partial^{2}\left(S_{B}^{\prime \prime }+S_{P}\right)}{\partial \tau^{\prime \prime }\partial x_{P}^{\prime \prime }} & 0 & \frac{\partial^{2}\left(S_{B}^{\prime \prime }+S_{P}\right)}{\partial {\tau^{\prime \prime }}^{2}} & \frac{\partial^{2}S_{P}}{\partial \tau^{\prime \prime }\partial \tau }\\ \frac{\partial^{2}S_{R}}{\partial \tau \partial x_{R}^{\prime }} & \frac{\partial^{2}S_{P}}{\partial \tau \partial x_{P}^{\prime }} & \frac{\partial^{2}S_{R}}{\partial \tau \partial x_{R}^{\prime \prime }} & \frac{\partial^{2}S_{P}}{\partial \tau \partial x_{P}^{\prime \prime }} & \frac{\partial^{2}S_{R}}{\partial \tau \partial \tau^{\prime }} & \frac{\partial^{2}S_{P}}{\partial \tau \partial \tau^{\prime \prime }} & \frac{\partial^{2}\left(S_{R}+S_{P}\right)}{\partial \tau^{2}} \end{array}\right|,$$

with $\left\{x_{R}^{\prime },x_{P}^{\prime },x_{R}^{\prime \prime },x_{P}^{\prime \prime },\tau^{\prime },\tau^{\prime \prime },\tau \right\}$ now denoting the index-one saddle point of $S^{\left(4\right)}$. We have again used Cauchy–Riemann identities to write derivatives of t in terms of derivatives of τ. As before, we evaluate the reactant partition function within the semiclassical approximation [18].

In this way, we have considerably simplified the exact quantum-mechanical expression for the three-state case (equation (4.4)), for which carrying out the double sum over the bridge states is tedious and numerically challenging due to the sum containing many terms of similar magnitude and opposite sign. By contrast, instanton theory defines a unique semiclassical pathway which provides a clear picture of the reaction mechanism.

Because the instanton trajectory travels below the bridge, it implies that the bridge states should be considered as *virtual* states. This is in conflict with the standard assumption that, because the bridge drops below $V_{\mathrm{RP}}$, it should be considered as an *intermediate* [12] which accepts population transfer in a sequential mechanism [49]. However, as we shall demonstrate below, the instanton formalism predicts the correct behaviour in the low-temperature limit, whereas an expression based on a sequential mechanism does not.

The instanton approach is, however, only applicable if a saddle point of the action exists, which limits the theory to the deep-tunnelling regime. This will always be the situation at low enough temperature, but above a certain crossover temperature, the saddle point will disappear and it is clear that a different theory will be required.

### (b) Application to tunnelling through quantum dots

Although we derived our semiclassical expressions with low-temperature bridge-mediated electron transfer in mind, the fourth-order rate theory is quite general and should be valid for any set of PESs that fit the description above. We shall illustrate the new theory using a simple one-dimensional system which models three quantum dots in a row with nearest-neighbour coupling. Tunnelling plays a key role in describing various useful properties of quantum dots that are used in single-electron transistors [98] and as qubits for quantum computers [99].

The Born–Oppenheimer formalism typically treats nuclear dynamics moving on a PES or PESs defined by integrating out the electronic degrees of freedom. However, for a simple description of quantum dots, one typically treats a single electron as the dynamical variable and constructs potential-energy surfaces which describe its interaction with the bulk semiconductor. The mathematical formalism is otherwise identical. For small diabatic couplings, the adiabatic PESs will approach each other near the crossing points, thus invalidating the Born–Oppenheimer approximation and requiring a non-adiabatic rate theory.

Bound electron states of a quantum dot are commonly modelled as harmonic potentials [100] and so we define

4.11 $$V_{R}(x)=\frac{1}{2}m\omega^{2}x^{2},\;V_{B}(x)=\frac{1}{2}m\omega^{2}{\left(x-x_{B}\right)}^{2}+\varepsilon_{B}\;\mathrm{and}\;V_{P}(x)=\frac{1}{2}m\omega^{2}{\left(x-x_{P}\right)}^{2}-\varepsilon_{P},$$

where x denotes the coordinate of the single electron. We choose parameters that correspond to a self-assembled InAs quantum dot [101] with effective electron mass $m=0.24\;m_{\text{e}}$ and vibrational energy spacing ℏω=0.08 eV, which for simplicity is chosen to be the same for all states. The bridge and product minima are located at $x_{B}=9\;\mathrm{nm}$ and $x_{P}=18\;\mathrm{nm}$, respectively, from the reactant, which corresponds to a typical length scale for quantum dots. The bias for the bridge state is set to $\varepsilon_{B}=0.35\;\mathrm{eV}$ while the bias of the product state, $\varepsilon_{P}$, is varied from 0 to 0.88 eV. Finally, we choose a temperature of 125 K for all our calculations and assume that the couplings $\Delta_{\mathrm{RB}}$ and $\Delta_{\mathrm{BP}}$ are independent of position.

For such a system, the bridge-mediated fourth-order rate describes a process called elastic cotunnelling [100,102], in which an electron tunnels from a source (R) to a drain (P) via the non-resonant *virtual* states of an intermediate quantum dot (B). These virtual states are higher in energy than the initial and final state, which themselves must be equal in energy for the reaction to take place. We can see that this exact condition is what is imposed by the single Dirac delta function in equation (4.4). At higher temperatures, a sequential process may become dominant, for which the instanton theory derived in this work is not applicable.

For the quantum-mechanical calculations, we use equation (4.4) and smear the delta function by replacing it with sin⁡(πE/ℏω)/πE. This accounts for the physical mechanism of dissipation in the wells and allows an incoherent rate constant to be defined in a one-dimensional system which would otherwise display coherent dynamics. The substitution is equivalent to truncating the integral of the correlation function to the range t∈[−π/ω,π/ω]. For simplicity, quantum-mechanical results were computed only for biases at which the reactant and product vibrational states matched exactly, for which the sinc function takes a value of ${\left(\hbar \omega \right)}^{-1}$, whereas it is 0 for any channel which does not exactly conserve energy.

**Figure 5.**
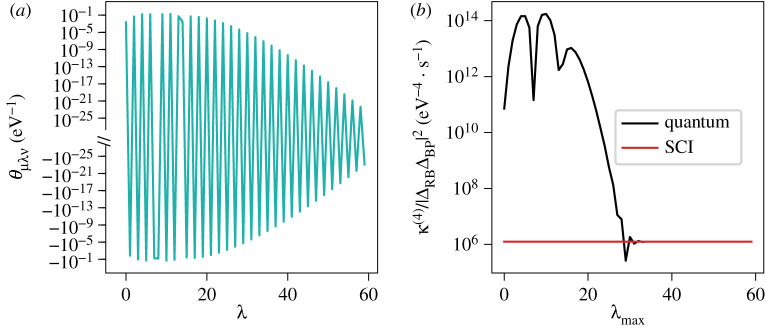
(*a*) The oscillations of the overlap $\theta_{\mu \nu \lambda }$ between the reactant state μ=0 and product state ν=2 through the bridge state λ, defined as $\theta_{\mu \lambda \nu }=\langle \chi_{R}^{\mu }|\chi_{B}^{\lambda }\rangle \langle \chi_{B}^{\lambda }|\chi_{P}^{\nu }\rangle {\left(E_{B}^{\lambda }-E_{R}^{\mu }\right)}^{-1}$, as a function of the bridge state λ. Lines connecting integer values of λ are used only to ease visualization. (*b*) The exact quantum-mechanical rate as a function of the maximum bridge level, $\lambda_{\text{max}}$, included in the sum. The SCI rate is shown as a horizontal line as the instanton formalism does not require a sum over bridge states. All calculations were done for the model system with a bias of $\epsilon_{P}=0.16\;\mathrm{eV}$. (Online version in colour.)

The quantum-mechanical calculations required a relatively large number of bridge states to converge because the overlap terms oscillate as functions of the bridge level, as shown in figure 5. The small magnitude of the values also makes computing the sum numerically challenging. For this particular model system, standard double-precision floating-point numbers were found to be adequate, but the nature of the sum means that one may require higher precision arithmetic in some cases.

For a particle in a harmonic potential, analytic expressions for the imaginary-time actions are known [6]. We use these results to evaluate the fourth-order instanton rates for our model system according to equation (4.10). According to the standard procedure, we use the semiclassical reactant partition function $Z_{R}={\left[2\sinh\frac{1}{2}\beta \hbar \omega \right]}^{-1}$, which happens to be exact for harmonic systems.

The instanton for the system with a moderate bias of $\varepsilon_{P}=0.08\;\mathrm{eV}$ is shown in figure 4*a*. Its energy lies well below the energy of any bridge state, suggesting that the largest contributions to the tunnelling rate are from the lower vibrational states of R. This is in agreement with the quantum-mechanical calculation, which indicates that the largest contribution comes from the vibrational ground state (≈50%), followed by the first excited state (≈40%).

**Figure 6.**
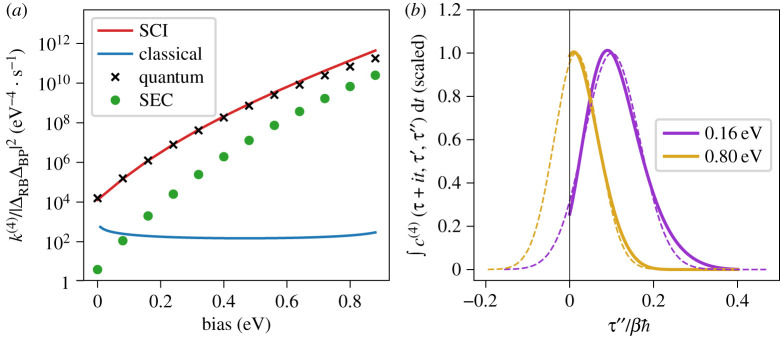
(*a*) Coupling-independent rates as a function of the bias, $\varepsilon_{P}$, using our new fourth-order instanton theory (equation (4.10)), the exact quantum-mechanical expression (equation (4.4)), the classical approximation (equation (4.14)), and the superexchange-mediated effective coupling approximation (equation (2.4a)) with effective coupling (equation (4.12)). (*b*) A plot showing how a steepest-descent integration over $\tau^{\prime \prime }$ overestimates the rate as a result of erroneously including the region $\tau^{\prime \prime }<0$. The two-time correlation function (defined via a steepest-descent integral of equation (4.7) over t) with $\tau^{\prime \prime }$ varied and $\tau^{\prime }$ fixed at the value at the stationary point is shown with a solid line and the corresponding Gaussian used by the steepest-descent approximation with a dashed line of the same colour. Each pair of lines of the same colour is scaled by its value at the stationary point. (Online version in colour.)

The calculated rates are plotted as a function of the bias, $\varepsilon_{P}$, in figure 6*a*. Because the rate constant is proportional to the current, these results are related to the current–voltage plots often studied in molecular electronics [103]. We see that the SCI and exact rates generally agree very well with each other, are within about 20% at low biases and differ by a factor of approximately 2 at the largest bias considered. This point corresponds to an instanton with an infinitesimally small trajectory on B and thus represents the limit of the method’s applicability. At stronger biases than this, a different SCI method would need to be derived, as the energies at the RB and BP hopping points ($V_{\mathrm{RB}}$ and $V_{\mathrm{BP}}$) would no longer be below $V_{\mathrm{RP}}$. The main cause of the error can be traced back to the steepest-descent integration over $\tau^{\prime }$ and $\tau^{\prime \prime }$ in equation (4.6). As illustrated in figure 6*b*, this overestimates the integral, particularly for high biases when the instanton has small values of $\tau^{\prime }=\tau^{\prime \prime }$.

#### (i) Comparison with superexchange-mediated effective-coupling approximation

A commonly used approximation to the fourth-order rate can be made by assuming that, relative to the large gap between reactant and bridge, one can ignore the vibrational energy-level structure of the bridge. All bridge states are thus assigned the same energy and the sums over the bridge levels in equation (4.4) can then be eliminated using the resolution of the identity. This gives an approach known as the superexchange-mediated effective coupling (SEC) approximation [49] and has a form equivalent to the second-order rate from R to P (equation (2.4a)) but with an effective coupling $\Delta_{\mathrm{RP}}^{\text{SEC}}$, that approximately accounts for the influence of the bridge

4.12 $$\Delta_{\mathrm{RP}}^{\text{SEC}}=\frac{\Delta_{\mathrm{RB}}\Delta_{\mathrm{BP}}}{E_{B}^{\left(0\right)}-E_{R}^{\left(0\right)}},$$

where $E_{R}^{\left(0\right)}$ and $E_{B}^{\left(0\right)}$ are the zero-point energies of the reactant and bridge. Just as for the exact fourth-order rate, we evaluate the expressions using a smeared delta function and a quantum-mechanical partition function as in §4(a).

From figure 6*a*, we observe that the SEC approximation significantly underestimates the rates when compared to the exact results and is much less accurate than the fourth-order instanton. This behaviour can be easily explained within the instanton formalism, which could in principle be applied on top of the SEC approximation. In this case, the dominant tunnelling pathway would travel only on R and P states, as in the golden-rule case. The total abbreviated action (equation (2.16c)) along the SEC pathway would be $W^{\left(2\right)}(E)=W_{R}+W_{P}$, which is necessarily greater than the fourth-order abbreviated action $W^{\left(4\right)}(E)=W_{R}+W_{B}^{\prime }+W_{P}+W_{B}^{\prime \prime }$ for a given energy, E, as the paths travelling on the bridge have a lower potential energy than they would in the reactant or product states. By the same argument, it can also be shown that $-\partial W^{\left(2\right)}/\partial E<-\partial W^{\left(4\right)}/\partial E$. It follows that the action $S^{\left(2\right)}=\min_{E}\{W^{\left(2\right)}(E)-\beta \hbar E\}$ must be greater than $S^{\left(4\right)}=\min_{E}\{W^{\left(4\right)}(E)-\beta \hbar E\}$ and that therefore the SEC approximation predicts that tunnelling occurs at a lower energy than necessary, with a larger action than in the fourth-order instanton theory, typically leading to an underestimation of the rate.

Note that there are various alternative options for how to define the effective coupling. In particular, one could enforce detailed balance using the denominator $E_{B}^{\left(0\right)}-\frac{1}{2}(E_{R}^{\left(0\right)}+E_{P}^{\left(0\right)})$ or $\sqrt{E_{B}^{\left(0\right)}-E_{R}^{\left(0\right)}}\sqrt{E_{B}^{\left(0\right)}-E_{P}^{\left(0\right)}}$ instead [49]. However, it is clear that any different choice simply shifts the whole curve, and although this may improve the results overall, it cannot be used to give uniformly accurate rates for both large and small biases.

#### (ii) Comparison with classical sequential mechanism

The expression for the classical rate of a one-dimensional bridge-mediated transfer in the limit of small couplings is [12]

4.13 $$k_{\text{cl}}^{\left(4\right)}Z_{R}^{\mathrm{cl}}=\frac{1}{2\pi \hbar }\int_{V^{\text{‡}}}^{\infty }\Gamma_{\mathrm{RB}}\;\Gamma_{\mathrm{BP}}\;e^{-\beta E}\;dE,$$

where $Z_{R}^{\mathrm{cl}}={\left(\beta \hbar \omega \right)}^{-1}$ is the classical partition function for the reactant and $V^{\text{‡}}$ is the higher of the reactant/bridge or product/bridge intersections, i.e. $\text{max}(V_{\mathrm{RB}},V_{\mathrm{BP}})$. The Landau–Zener [44,45] hopping probability in the non-adiabatic limit is $\Gamma_{\mathrm{RB}}=4\pi |\Delta_{\mathrm{RB}}{|}^{2}/\hbar \kappa_{\mathrm{RB}}v_{\mathrm{RB}}(E)$ (which includes a factor of 2 to account for the second crossing of nonreactive paths [12]), where $\kappa_{\mathrm{RB}}=|\nabla V_{R}(x_{\mathrm{RB}})-\nabla V_{B}(x_{\mathrm{RB}})|$ is the difference in the slopes and $v_{\mathrm{RB}}(E)=\sqrt{2(E-V_{\mathrm{RB}})/m}$ is the classical velocity at the reactant/bridge crossing point, $x_{\mathrm{RB}}$. $\Gamma_{\mathrm{BP}}$ is defined analogously for the product/bridge intersection.

Assuming $V_{\mathrm{RB}}>V_{\mathrm{BP}}$, the integral can be evaluated analytically to give

4.14 $$k_{\text{cl}}^{\left(4\right)}Z_{R}^{\mathrm{cl}}=\frac{4\pi m|\Delta_{\mathrm{RB}}\Delta_{\mathrm{BP}}{|}^{2}}{\hbar^{3}\kappa_{\mathrm{RB}}\kappa_{\mathrm{BP}}}K_{0}(\frac{1}{2}\beta (V_{\mathrm{RB}}-V_{\mathrm{BP}}))\;e^{-\beta (V_{\mathrm{RB}}+V_{\mathrm{BP}})/2},$$

where $K_{0}$ is the zeroth modified Bessel function of the second kind. Figure 6*a* shows how the classical rate varies with respect to the bias for the model system and we see that it fails to predict the trend of the rate with respect to the product bias. Note also that the expression in equation (4.14) spuriously tends to infinity when the crossing point energies $V_{\mathrm{RB}}$ and $V_{\mathrm{BP}}$ are equal.

Using the asymptotic relation $K_{0}(z)∼\sqrt{\pi /2z}\;e^{-z}$ as z→∞, we can gain some useful insights into the mechanism underlying the classical rate. When the quantity $\beta (V_{\mathrm{RB}}-V_{\mathrm{BP}})$ is large (i.e. for low temperatures and strong exothermic bias), the rate tends to an expression with the exponential term $e^{-\beta V_{\mathrm{RB}}}$, and we identify $V_{\mathrm{RB}}$ as the activation energy. In fact, the expression reduces in this limit to the classical second-order rate (equation (2.23)) (for a reaction from R to B) with a classical reactant partition function and an effective coupling given by

4.15 $$|\Delta_{\text{eff}}{|}^{2}=\frac{\sqrt{8m}\pi |\Delta_{\mathrm{RB}}\Delta_{\mathrm{BP}}{|}^{2}}{\hbar \kappa_{\mathrm{BP}}\sqrt{V_{\mathrm{RB}}-V_{\mathrm{BP}}}}.$$

At the relatively low temperature of our problem, for which $\beta \approx 90\;{\mathrm{eV}}^{-1}$, this limit is valid across all but the smallest exothermic biases considered. As for the SEC approximation, the classical sequential mechanism leads to a rate expression which can be written as the second-order rate with an effective coupling. However, in contrast to the SEC approximation, the effective second-order rate corresponds to a transfer from R to B instead of R to P and thus has an activation energy which is independent of exothermic bias. This explains why the predictions are contrary to the quantum-mechanical benchmark.

## 5. Conclusion

In this paper, we have explained how SCI theory can be generalized to study tunnelling effects in certain non-adiabatic chemical reactions. In particular, we have reviewed its application in the golden-rule limit, derived new closed-form expressions in separable cases and demonstrated that it can give accurate results where simpler methods based on global linear approximations fail. It will be interesting to see if the predictions of instanton theory will be significantly different from those presented in the literature and whether better agreement with experiment can be achieved in this way.

We have presented the working equations to evaluate golden-rule instanton theory within the ring-polymer formalism. In particular, we demonstrate how to treat translational and rotational degrees of freedom in a manner applicable to molecular systems in the gas phase. These methods are currently being applied to various molecular systems in our group and results will be published in forthcoming work.

Finally, we have generalized instanton theory beyond the golden-rule limit to treat the rate of a fourth-order reaction proceeding from reactants to products via a bridge. Unlike expressions based on the classical sequential mechanism, instanton theory was able to correctly predict the dependence of the rate on the bias using a deep-tunnelling pathway under the bridge. It gave good quantitative agreement with benchmark quantum-mechanical calculations and was significantly more accurate than the superexchange-mediated effective coupling approximation. It would be trivial to extend the approach for cases with multiple intermediate bridges. However, further extensions will be necessary to treat systems in the inverted regime or with high-energy bridges.

The nature of the systems for which this fourth-order instanton theory is applicable requires long tunnelling pathways. These will only exist at low temperature and for light tunnelling particles. For this reason, we chose to demonstrate the method using tunnelling of an electron through a row of quantum dots. It is clear that an accurate treatment of deep tunnelling is essential for this problem, as the classical mechanism fails to predict the correct trends.

In this work, we have applied the new fourth-order instanton approach only to a one-dimensional harmonic model system for which the propagators are known analytically. The ring-polymer approach used successfully for the second-order golden-rule case can, however, be directly generalized to the fourth-order rate, which would make this method applicable to complex multidimensional anharmonic systems.

## Figures and Tables

**Figure 7.**
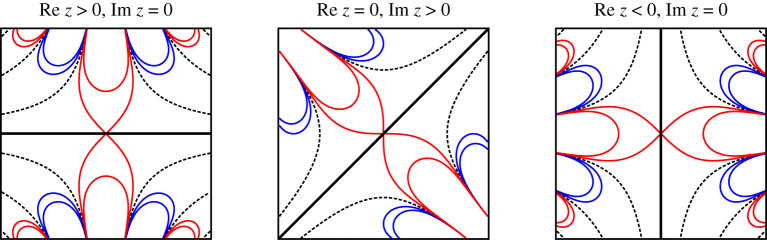
Real part of the integrand in (A5) multiplied by the phase of the line element along the direction of steepest descent (thick black line). The horizontal and vertical axes correspond to the real and imaginary parts of the coordinate y. The blue, red and dashed black contours correspond to negative, positive and zero values of the integrand, respectively. (Online version in colour.)

## Data Availability

This article has no additional data.

## Footnotes

1 The disadvantage of this approach is that instanton theory is not directly applicable to liquid systems [18]. It nonetheless plays a key role in the development of other path-integral methods which can describe fluxional environments [19,23–25].

2 For the application of path-integral methods to the inverted regime, see appendix A.

3 The formulation is equally valid in the endoergic inverted regime where $\tau_{R}<0$ and the index of the saddle point is $N_{R}f+1$, or $N_{R}f/2+1$ for a half-ring polymer.

## Appendix A. Propagators at negative imaginary times

The quantum-mechanical imaginary-time propagator can be written in the eigenbasis of the Hamiltonian ${\hat{H}}_{n}$,

A 1 $$K_{n}(x^{\prime \prime },x^{\prime },\tau_{n})=\sum_{\nu }e^{-\tau_{n}E_{n}^{\nu }/\hbar }\langle x^{\prime \prime }|\chi_{n}^{\nu }\rangle \langle \chi_{n}^{\nu }|x^{\prime }\rangle ,$$

and the flux autocorrelation function (equation (2.7)) can similarly be written as

A 2 $$c^{\left(2\right)}(\tau )=\sum_{\mu \nu }|\Delta_{\mathrm{RP}}^{\mu \nu }{|}^{2}\;e^{-(\beta \hbar -\tau )E_{R}^{\mu }/\hbar }\;e^{-\tau E_{P}^{\nu }/\hbar },$$

where for brevity we have taken t=0. Using the relations $\tau_{R}=\beta \hbar -\tau$ and $\tau_{P}=\tau$, both sums are absolutely convergent [104] when 0<τ<βℏ, and, while the right-hand side of equation (A1) for n=P diverges when τ<0, equation (A2) remains well-defined, at least in some cases [42]. This implies the existence of an analytic continuation of equation (A1) that is also valid at negative imaginary time, as we will now show. First, consider the short-time propagator [6]

A 3 $$K_{n}(x^{\prime \prime },x^{\prime },z)∼{\left(\frac{m}{2\pi \hbar z}\right)}^{f/2}\exp\left\{-\frac{1}{\hbar }\left[\frac{m\|x^{\prime \prime }-x^{\prime }{\|}^{2}}{2z}+zV_{n}\left(\frac{x^{\prime }+x^{\prime \prime }}{2}\right)\right]\right\}\;(z\to 0),$$

where we replace $\tau_{n}$ with z to indicate that the time variable can be complex. The expression is singular at z=0, which explains why its series representation in equation (A1) diverges for $\tau_{n}<0$ [105]. Crucially, equation (A3) is defined for any z, not just Re z>0 and hence constitutes the short-time limit of the desired analytic continuation. To go beyond the short-time limit, consider the composition property of the propagator [6],

A 4 $$K_{n}(x^{\prime \prime },x^{\prime },z^{\prime \prime }+z^{\prime })=\int dx\;K_{n}(x^{\prime \prime },x,z^{\prime \prime })K_{n}(x,x^{\prime },z^{\prime }),$$

where the integration contour is defined such that the relation holds. For simplicity, we set $z^{\prime }=z^{\prime \prime }=z/2$. For Re z>0, the standard integration bounds of ±∞ are suitable, but for Re z<0, we must find an alternative. In what follows we take z→0, and consider $\left\|x^{\prime \prime }-x^{\prime }\|∼O(z^{1/2}\right)$. The latter is imposed because $K_{n}(x^{\prime \prime },x^{\prime },z)$ will shortly appear in an integral for which contributions from larger displacement amplitudes are vanishingly small in the specified limit (see ch. 7.3 of [6]). Under these conditions, equation (A4) implies

A 5 $$\begin{array}{rl} K_{n}(x^{\prime \prime },x^{\prime },z) & \;∼{\left(\frac{m}{2\pi \hbar z}\right)}^{f/2}\exp\left\{-\frac{1}{\hbar }\left[\frac{m\|x^{\prime \prime }-x^{\prime }{\|}^{2}}{2z}+zV_{n}\left(\frac{x^{\prime }+x^{\prime \prime }}{2}\right)\right]\right\}\\ & \;\times \int dy\;{\left(\frac{2m}{\pi \hbar z}\right)}^{f/2}\exp\left[-\frac{2m}{\hbar z}\|y{\|}^{2}\right]\;(z\to 0), \end{array}$$

where

A 6 $$y=x-\tilde{x},\;\tilde{x}=\frac{x^{\prime }+x^{\prime \prime }}{2}-\frac{z^{2}\nabla V_{n}((x^{\prime }+x^{\prime \prime })/2)}{8m}.$$

Equations (A3) and (A5) agree if integration is performed along the direction of steepest descent in the complex y plane (figure 7), which for Re z<0, Im z=0, lies along the imaginary axis.

It follows that the correct path-integral representation for a propagator at $\tau_{n}<0$ is

A 7 $$\begin{array}{rl} K_{n}(x^{\prime \prime },x^{\prime },\tau_{n}) & \;=\lim_{N_{n}\to \infty }\prod_{i=1}^{N_{n}-1}\left[\int_{-i\infty +{\tilde{x}}_{i}}^{i\infty +{\tilde{x}}_{i}}\frac{dx_{i}}{A}\right]\\ & \;\exp\left\{-\frac{1}{\hbar }\left[\sum_{i=0}^{N_{n}-1}\frac{m\|x_{i+1}-x_{i}{\|}^{2}}{2\epsilon_{n}}+\epsilon_{n}V_{n}\left(\frac{x_{i}+x_{i+1}}{2}\right)\right]\right\}, \end{array}$$

where $\epsilon_{n}=\tau_{n}/N_{n}$, $x_{0}\equiv x^{\prime }$, $x_{N_{n}}\equiv x^{\prime \prime }$, and $A=i^{f}{\left(2\pi \hbar |\epsilon_{n}|/m\right)}^{f/2}$. The sum in square brackets is the discretized representation of the Euclidean action in equation (2.9), and the points ${\tilde{x}}_{i}$ are the coordinates $x(i\epsilon_{n})$ along the trajectory that extremizes it. These coordinates generalize the single stationary point $\tilde{x}$ in (A6). Since this stationary point is a maximum along the real axis it follows that ${\tilde{x}}_{i}$ *maximizes* the action $S_{n}(x^{\prime \prime },x^{\prime },\tau_{n})$ when $\tau_{n}<0$, which can also be deduced from the relation $S_{n}(x^{\prime \prime },x^{\prime },-\tau_{n})=-S_{n}(x^{\prime \prime },x^{\prime },\tau_{n})$ [42]. Therefore, the same trajectory extremizes $S_{n}(x^{\prime \prime },x^{\prime },\pm \tau_{n})$, but is a minimum for positive imaginary time and a maximum for negative imaginary time. Hence the semiclassical approximation to the propagator, obtained by steepest-descent integration [64], is

A 8 $$K_{n}(x^{\prime \prime },x^{\prime },\pm |\tau_{n}|)∼\sqrt{\frac{\left|C_{n}\right|}{{\left(2\pi \hbar \right)}^{f}}}\;e^{\mp S_{n}(x^{\prime \prime },x^{\prime },|\tau_{n}|)},$$

where $\left|C_{n}\right|$ is the absolute value of the f×f determinant (equation (2.10)). To complete the derivation, we substitute this result into equation (2.7) and integrate over the end-points. Once again the integration contour is chosen along the direction of steepest descent, leading to a correlation function that has the correct high-temperature limit, as shown in [41], and is well defined for both the normal and inverted regimes. For the latter, steepest descent is along the imaginary axis for f degrees of freedom, which are therefore maxima of the total action in the space of real coordinates. Combining this with our earlier result and noting that the stationary action is a maximum of τ, we see that in the inverted-regime the instanton is an index-$\left(N_{P}f+1\right)$ saddle point of the total action in the space of real discretized trajectories and imaginary time, as claimed in §3.
